# Examining the impact of larval source management and insecticide-treated nets using a spatial agent-based model of *Anopheles gambiae* and a landscape generator tool

**DOI:** 10.1186/1475-2875-12-290

**Published:** 2013-08-21

**Authors:** SM Niaz Arifin, Gregory R Madey, Frank H Collins

**Affiliations:** 1 Department of Computer Science and Engineering, University of Notre Dame, Notre Dame, 46556, IN, USA; 2 Department of Biological Sciences, 100 Galvin Life Sciences Center, University of Notre Dame, Notre Dame, 46556, IN, USA

**Keywords:** Malaria, Agent-based model, Spatial model, *Anopheles gambiae*, Replication, Integrated vector management, Larval source management, Insecticide-treated nets, Combined interventions, Landscape generator

## Abstract

**Background:**

Agent-based models (ABMs) have been used to estimate the effects of malaria-control interventions. Early studies have shown the efficacy of larval source management (LSM) and insecticide-treated nets (ITNs) as vector-control interventions, applied both in isolation and in combination. However, the robustness of results can be affected by several important modelling assumptions, including the type of boundary used for landscapes, and the number of replicated simulation runs reported in results. Selection of the ITN coverage definition may also affect the predictive findings. Hence, by replication, independent verification of prior findings of published models bears special importance.

**Methods:**

A spatially-explicit entomological ABM of *Anopheles gambiae* is used to simulate the resource-seeking process of mosquitoes in grid-based landscapes. To explore LSM and replicate results of an earlier LSM study, the original landscapes and scenarios are replicated by using a landscape generator tool, and 1,800 replicated simulations are run using absorbing and non-absorbing boundaries. To explore ITNs and evaluate the relative impacts of the different ITN coverage schemes, the settings of an earlier ITN study are replicated, the coverage schemes are defined and simulated, and 9,000 replicated simulations for three ITN parameters (coverage, repellence and mortality) are run. To evaluate LSM and ITNs in combination, landscapes with varying densities of houses and human populations are generated, and 12,000 simulations are run.

**Results:**

General agreement with an earlier LSM study is observed when an absorbing boundary is used. However, using a non-absorbing boundary produces significantly different results, which may be attributed to the unrealistic killing effect of an absorbing boundary. Abundance cannot be completely suppressed by removing aquatic habitats within 300 m of houses. Also, with density-dependent oviposition, removal of insufficient number of aquatic habitats may prove counter-productive. The importance of performing large number of simulation runs is also demonstrated. For ITNs, the choice of coverage scheme has important implications, and too high repellence yields detrimental effects. When LSM and ITNs are applied in combination, ITNs’ mortality can play more important roles with higher densities of houses. With partial mortality, increasing ITN coverage is more effective than increasing LSM coverage, and integrating both interventions yields more synergy as the densities of houses increase.

**Conclusions:**

Using a non-absorbing boundary and reporting average results from sufficiently large number of simulation runs are strongly recommended for malaria ABMs. Several guidelines (code and data sharing, relevant documentation, and standardized models) for future modellers are also recommended.

## Background

Vector management, involving a wide array of interventions, is the primary means of malaria prevention and control in Africa [1,2]. Malaria modelling, both mathematical and agent-based, can play important roles to quantify the effects of malaria-control interventions and to answer other interesting research questions. Models can play key roles in selecting appropriate combinations of interventions to interrupt transmission and in setting response timelines and expectations of impact. Mathematical modelling of malaria transmission dates back to the early models of Ross and Macdonald [3,4]. Recent mathematical models include a dynamic model of Smith and McKenzie [5], a weather-driven parasite dynamics transmission model of Hoshen and Morse [6], an individual-based model of Depinay *et al.*[7], the OpenMalaria epidemiology model [8,9], an intervention-based model of Yakob and Yan [2] and others.

Agent-based models (ABMs) of malaria have also been used to model the basic behaviour of individual mosquitoes, including interactions within agents and to their environment. These interactions, involving a large number of agents, provide the opportunities to explore interesting emerging phenomena, such as population-level characteristics. Recent malaria ABMs include models of Gu and Novak [10,11], a transmission-directed model of Eckhoff [12] and an individual-based simulation model of Griffin *et al.*[13]. A summary comparing model features from some recent malaria models is given in Table 1.

**Table 1:** Summary of feature comparisons from several malaria models, including new features modelled by this study

**Model feature**	**Malaria models**				
	**Gu** &**Novak**[10,11]	**Yakob** &**Yan**[2]	**Eckhoff**[12]	**Chitnis *et al.***[8]	**This study**
Model type	agent-based	mathematical	individual-based	mathematical	agent-based
Spatial representation	landscape-based	N/A	space can be represented as a lattice of points	N/A	landscape-based
Automation of landscape generation (e.g., using separate tools) *	no	N/A	no	N/A	yes (*VectorLand*)
Boundary type of landscape *	absorbing	N/A	N/A	N/A	non-absorbing
Average of multiple simulations *	no	no	no	no	yes
Time-step resolution	daily	daily	hourly	daily	hourly
Age-specific mortality	no	no	N/A	no	yes (see [19,21])
Daily mortality rate (immature stages)	fixed, 0.2	fixed, 0.15	temperature-dependent	N/A	age-specific (for larvae)
Daily mortality rate (adult stages)	fixed, 0.2	fixed, 0.15	adult life expectancy of 10 days	N/A	age-specific
Fecundity (eggs/oviposition)	fixed, 80	N/A	fixed, 100	N/A	*N* (170*,* 30)
Variability in daily temperature	no	no	yes	yes	yes (25°C)
Length of individual simulation run	200 days for LSM, 300 days for ITNs	N/A	> 6 years	N/A	1 year
Interventions modelled	LSM, ITNs	LSM, ITNs	IRS, ITNs, larvicides, space spraying	ITNs, IRS	LSM, ITNs
Time-step of intervention application	day 100 for LSM, day 150 for ITNs	N/A	N/A	N/A	day 100
Explores combined interventions	no	yes	yes	yes	yes
Variability in human population	no	yes	no	yes	yes
Coverage scheme used for ITNs *	proportion of house-holds with bed nets	proportion of populations sleeping under bed nets	proportion of populations sleeping under bed nets	proportion of populations sleeping under bed nets	partial and complete coverage (see Methods)
Comparison of coverage schemes for ITNs *	no	no	no	no	yes

Each row represents a specific model feature. Each column represents a specific malaria model. Features marked with * are either modelled with extensions, or may be treated as new (not modelled earlier by other studies). Text in the cells represent whether the feature is implemented/available in the model, including simple yes/no, or other comments. N/A means *not applicable* or *not available*. Time-step resolution indicates the most fine-grained time resolution (for example, models reported as hourly are also capable of reporting events on a *daily* basis). For some features, whether the model implements variability of the feature is stated (e.g., variability in daily temperature), and the default value used in the model is noted in parentheses. For fecundity, *N* indicates a normal distribution with *mean* and *standard deviation*. Daily mortality and age-dependent mortality refer to mortality of mosquitoes.

The *Anopheles* mosquitoes need to access blood meals and aquatic oviposition sites to complete their life cycle. Availability of these *ecological resources*, i.e., the human houses and aquatic habitats, has long been recognized as a crucial determinant of malaria transmission [3]. Reduced availability of either type of these spatial resources would prolong the gonotrophic cycle of the female mosquito and potentially affect malaria transmission. Also, these resources define landscape features such as spatial heterogeneity, host availability, etc., the importance of which for vector control have been demonstrated by several studies. For example, using an availability-based model, Killeen *et al.* showed the influence of host availability on malaria vectors in African communities [14]. Menach *et al.* showed how the heterogeneity in human biting reflects the underlying spatial heterogeneity in the attractiveness, distribution and suitability of human houses and aquatic habitats [15]. To demonstrate the spatial characteristics of transmission by the *Anopheles gambiae* complex in sub-Saharan Africa, Carter *et al.* identified some breeding sites as the foci of transmission, which are closely associated with particular locations; and the non-random distribution (clustering) of malaria case incidences in different households [16]. Conclusions from the above studies naturally lead to habitat-based interventions, which necessitates a landscape approach to incorporate the spatial processes of mosquito foraging for oviposition and host-seeking [17]. Spatially-explicit models, which permit the refined characterization of resource seeking to predict the impact of habitat-based interventions, can prove valuable to this end [10,11,17]. Earlier, an ABM of malaria, derived from a conceptual entomological model of the *An. gambiae* life cycle, was developed [18]. The model was later extended to have explicit spatial representation [19,20]. The ABM is presented here as a runnable program (JAR file as Additional file 1), with a sample input file (as Additional file 2), respectively.

Larval source management (LSM), insecticide-treated nets (ITNs) and indoor residual spraying (IRS) have been extensively used as intervention tactics to reduce and control malaria in sub-Saharan Africa. Impacts of various interventions (including LSM, ITNs and IRS) have been investigated by early and recent studies [2,8,10-12,21-23]. LSM (also known as source reduction), one of the oldest tools in the fight against malaria, refers to the management of aquatic habitats in order to restrict the completion of immature stages of mosquito development. In a recent study, Fillinger and Lindsay suggest that LSM can be successfully used for malaria control in African transmission settings by highlighting historical and recent successes, and discuss its potential in an integrated vector management (IVM) approach working towards malaria elimination [24,25]. In areas with moderate and focal malaria transmission where larval habitats are accessible and well-defined, LSM is also cost-effective when compared with IRS and LLINs [26]. For this study, LSM refers to the permanent elimination of targeted aquatic habitats, which may be achieved by various methods that include landscaping, drainage of surface water, land reclamation and filling, coverage of large water storage containers, wells and other potential breeding sites, etc. [25].

ITNs, particularly the long-lasting insecticidal nets (LLINs), are considered among the most effective vector control strategies currently in use [2,27-29]. To combat against the major malaria vectors (including *An. gambiae*) in Africa, scale-up applications of ITNs, which can offer direct personal protection to users as well as indirect, community protection to non-users (through insecticidal and/or repellent effects), are advocated [11,28]. Primarily due to mathematical convenience, earlier models that studied the impact of ITNs on malaria transmission assumed a uniform contact structure between mosquitoes and hosts across the landscape [30,31]. However, empirical data indicating limited flight ranges and sensory perception of mosquitoes suggest that proximity between the mosquitoes and their hosts can play a crucial role in the mosquito biting behaviour [32-36]. Hence, spatially-explicit models are needed to analyse the local host-seeking process of the mosquitoes, and to study the responses of mosquitoes to ITNs. Such models can also provide evidence for the need of entomological surveillance for evaluation of scale-up ITN programmes [11].

Replicability of the *in-silico* experiments and simulations performed by various malaria models bear special importance. Although computational science has led to exciting new developments, the nature of the work has also exposed shortcomings in the general ability of the research community to evaluate published findings [37]. Replication, which is treated as the scientific gold standard to judge scientific claims, allows independent researchers to address a scientific hypothesis and produce evidence *for* or *against* it [37,38]. Replication confirms reproducibility, which refers to the independent verification of prior findings, and is at the core of the spirit of science [39,40]. In agent-based modelling and simulation (ABMS), replication is also known as model-to-model comparison, alignment, or cross-model validation. It falls under the broader subject of verification and validation (V&V). One of its goals is to try to align multiple models in order to investigate whether they produce similar results [41,42]. When the original models (e.g., the source codes) are available, a stricter form of model verification, known as *docking*, may also be performed. In the past, the process of achieving a complete dock between separate implementations of the malaria ABMs was shown [19,20].

One of the goals of this study is to replicate the results and extend some assumptions of two published studies performed by the same authors. These studies explore the impact of applying LSM and ITNs as stand-alone interventions using an ABM [10,11] (for brevity, the studies are hereafter referred to as GN-LSM and GN-ITN, and the ABM used as GN-ABM, where GN refer to the initials of last names of the authors). Critical examination of these studies reveals that although they provide reasonably plausible results, two major assumptions may be extended regarding: (1) the number of replicated simulation runs, and (2) the boundary type of the landscapes.

Any simulation model which involves substantial stochasticity should conduct sufficient number of replicated runs (with identical parameter settings but different random seeds), and the average and/or aggregate results of these replicated runs should be reported, as opposed to reporting results from a single run. Sufficient number of replications is required to ensure that, given the same input, the average response can be treated as a deterministic number, and not as random variation of the results. This allows to obtain a complete statistical description of the model variables. The same principle also applies to a set of stochastic (Monte Carlo) simulation models in other domains (e.g., traffic flow, financial problems, risk analysis, supply chain forecasting, etc.), where, in most cases, the standard practice is to report the averages and standard deviations of the measures of interest (known as the *Measures of Effectiveness*, or MOEs) [43,44].

Since most epidemiology models (including ABMs) involve substantial stochasticity in the forms of probability-based distributions and equations, performing sufficient number of replicated runs is also important for validation of the results. In malaria ABMs, decisions are often simulated using random draws from certain distributions. These sources of randomness are used to represent the diversity of model characteristics, and the behaviour uncertainty of the agents’ actions, states, etc., with the goal to mimic/simulate the reality as closely as desired. For example, in the ABM, when a host-seeking mosquito searches for a blood meal in a ITN-covered house, a 50% ITN mortality would mean that it may die with a probability of 0.5, which can be simulated using random draws from a *uniform* distribution. As another example, the number of eggs in each egg-batch of a *Gravid* mosquito is simulated using random draws from a *normal* distribution with *mean (average)* = 170 and *standard deviation* = 30. The randomness has significant impact on the results of the simulation, and different simulation runs can therefore produce significantly different results, due to a different sequence of pseudo-random numbers drawn from the distributions. So, replicated runs for all simulations reported in this study are performed, as opposed to single runs performed in GN-LSM and GN-ITN [10,11].

The second issue, the use of a specific boundary type, may greatly impact the mosquito movement process. In general, three different boundary types are commonly used in ABMS: absorbing, non-absorbing and reflecting. With an *absorbing* boundary, mosquitoes are permanently removed (effectively killed) when they hit an edge of the landscape’s boundary. On the other hand, with a *non-absorbing* boundary, when mosquitoes hit an edge, they re-enter the landscape from the edge directly opposite of the exiting edge (and thus are not killed due to hitting the edge). Unless the underlying landscape reflects a completely isolated geographic location (e.g., an island far away from the mainlands), in reality, when mosquitoes hit an edge, logical approaches are either to reflect the mosquito back from the same edge (reflecting boundary), or to coerce the mosquito to re-enter from the opposite edge (non-absorbing boundary). However, a non-absorbing boundary may more realistically capture the mosquito population dynamics. This is especially true when the resource densities are high and the resources are more evenly distributed across the landscape. The GN-ABM uses an absorbing boundary for all landscapes. In this study, all landscapes are modelled topologically as 2D torus spaces (a 2D torus is a geometrical surface of revolution generated by revolving a circle in two-dimensional space about an axis coplanar with the circle; in ABM, a toroidal space resembles a donut topology, allowing an agent to re-enter the space from the opposite edge when it moves off one edge), and use a non-absorbing boundary. However, to compare with GN-LSM [10], results that use an absorbing boundary are reported first.

In malaria literature, multiple definitions of the term *ITN coverage* can be found. The Roll Back Malaria (RBM) Partnership uses ITN coverage as the proportion of households owning a bed net or sleeping under a bed net [45] (this definition is also used by GN-ITN [11]). On the other hand, the World Health Organization (WHO) reports ITN coverage as the number of bed nets distributed per person at risk [46]. In some studies, ITN coverage is also defined as the proportion of populations sleeping under treated bed nets [30], and is used more widely in recent models [2,8,12,30]. However, this distinction in multiple definitions of ITN coverage, primarily concerning coverage levels of households and individuals, has not been addressed (within a single study) by most recent models. The WHO emphasizes the importance of scale-up ITNs coverage beyond vulnerable population (children under five years of age and pregnant women) as a priority for combating malaria in tropical Africa [47]. Also, several studies have shown that the patterns of coverage and effective coverage are important determinants of ITN/LLIN success [13], and simple ITN/LLIN models in which the coverage scheme is not carefully designed can lead to overly optimistic results [31,48,49]. Thus, simulating different definitions of ITN coverage and assessing their relative impacts are important, especially when replicating and validating results of an earlier model that used either of these definitions (e.g., [11]). Hence, as an extension to GN-ITN [11], three different definitions/schemes of ITN coverage, which differ by the number of persons actually covered by bed nets in a ITN-covered house, are simulated and compared: 1) household-level *partial* coverage with *single* chance for host-seeking, 2) household-level *partial* coverage with *multiple* chances for host-seeking and 3) household-level *complete* coverage. All schemes are described in details in Methods (for the purposes of this study, coverage means access to an ITN; however, as described in Methods, household-level coverage and population-level coverage are defined as the proportion of the houses with coverage and the proportion of the people sleeping under ITNs, respectively).

A landscape generator tool, *VectorLand*, is also developed to aid in generating landscapes with varying spatial heterogeneity of both types of resources. An earlier version of *VectorLand* appeared in [19]. Here, a runnable program (in a JAR file) is presented as Additional file 3. It is emphasized that *VectorLand* is a tool to generate landscapes, which are then used as spatial input to the ABM; and is not a model in itself. A screenshot of *VectorLand* is given in Additional file 4.

There is now a consensus that malaria elimination with current tools is far more likely if the best available tools are used in combinations [27]. The IVM approach, promoted by the WHO, is a rational decision-making process for the optimal use of resources and efficient management for vector control. It actively considers the notion whether multiple interventions can be combined to control vector-borne diseases [25]. Because of improved efficacy, cost-effectiveness, ecological soundness and sustainability, IVM is increasingly being recommended as an option for sustainable malaria control [50]. The rationale of using combined interventions is that multiple interventions can offer synergistic effects on top of individual impacts offered by each intervention (when applied alone), thus producing a result that is greater than the sum of their individual effects. Such synergistic effects have been demonstrated by several model-based and field-based studies (if such synergy exists, it would be useful to understand and verify it in the field, and this study may prove helpful to this regard). Using a mathematical model, Yakob and Yan theoretically examined the application of LSM with ITNs in reducing malaria transmission [2]. The combined impact of ITNs (or LLINs) and IRS is examined by Chitnis *et al.* using the OpenMalaria model [8] and by a recent field-based study in south eastern Tanzania by Okumu *et al.*[51]. Using an ecological model, White *et al.* explored the impact of LLINs, IRS, larvicide and pupacide [52]. Eckhoff used a cohort-based vector simulation model to demonstrate the effects of increasing coverage with perfect IRS, combining IRS and ITNs, and combining larval control (using larvicides) and space spraying [12]. Using an individual-based simulation model with different combinations of LLINs, IRS, artemisinin-combination therapy (ACT), mass screening and treatment (MSAT) and vaccines, Griffin *et al.* showed that the combined interventions can result in substantial declines in malaria prevalence across a wide range of transmission settings [13]. Kleinschmidt *et al.* presented a summary of studies comparing the effect of IRS combined with ITNs [53]. Some of these studies suggest that when combined interventions are applied, it may be more beneficial to target different stages of the mosquito’s life cycle, rather than applying interventions that may interfere with each other (e.g., LLINs and IRS) [52].

Two important notions emerged from the conclusion of these studies: (1) when combined interventions are applied, the individual efficacy of each intervention needs to be ensured and (2) attacking different behaviours or life cycle stages of the mosquito may be more synergistic. Based on these, LSM and ITNs are selected, and their combined impacts are explored with the ABM. To ensure (1), the impacts of both are first examined as stand-alone interventions. In doing so, the two GN studies [10,11] are replicated, and some of the original assumptions are extended. It is interesting to note that no ABMs ever explored the combined impact of LSM and ITNs before (although some other combinations were explored using ABMs). Since LSM and ITNs primarily affect two different life cycle stages (i.e., larval and adult stages, respectively) and involve two different types of *ecological resources* (i.e., aquatic habitats and human houses, respectively), this combination is potentially important.

In this study, using the spatial ABM, the effects of LSM and ITNs are first investigated separately (in isolation), and then are compared to the results reported by the original studies [10,11] (the goal of replication is to achieve a qualitative (not absolute) match between results of the ABM and those reported in GN-LSM [10] and GN-ITN [11]). Then, using different population profiles to explore the human density effect, the combined impact of LSM and ITNs are investigated, and similar results reported by Yakob and Yan [2] are also discussed. Lastly, some guidelines for future ABM modellers, summarizing the insights and experience gained from this work of replicating the original models, are recommended. A systematic comparison of some features and assumptions of several recent malaria models, including those that are extended, or modelled for the first time by this study, is given in Table 1.

## Methods

**Figure 1:**
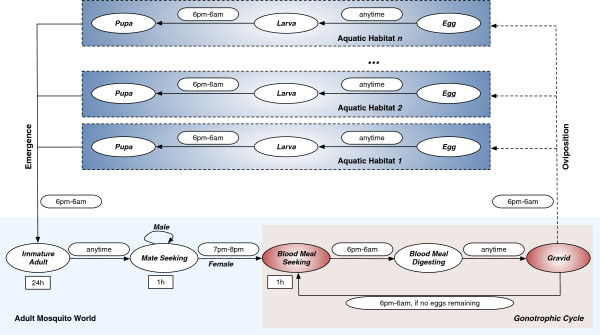
**Life cycle of mosquito agents (both males and females) in the ABM.** Each oval represents a state in the model. States in which agents move through the landscape are marked in red. The rectangles represent durations for the fixed-duration states. The symbol *h* denotes hour. Permissible time transition windows (from one state to another) are shown next to the corresponding state transition arrows as rounded rectangles. For example, the rounded rectangle labelled as “7pm-8pm” (between *Mate Seeking* and *Blood Meal Seeking*) indicates that female mosquitoes may transition from the *Mate Seeking* state to the *Blood Meal Seeking* state only during 7pm-8pm of each (simulated) day. Note that adult males, once reaching the *Mate Seeking* state, remain forever in that state until they die; adult females cycle through obtaining blood meals (in *Blood Meal Seeking* state), developing eggs (in *Blood meal Digesting* state), and ovipositing these eggs (in *Gravid* state) until they die.

For this study, an extended version of an agent-based entomological model of *An. gambiae* developed earlier is used. Detailed descriptions of the ABM, including the origin and implementation details of its core biological concepts as well as the functional forms, have been reported elsewhere [18-20]. The life cycle of mosquito agents in the ABM, reproduced and slightly edited from [19], is shown in Figure 1.

### Movement of adult mosquitoes

In the spatial ABM, movement of female mosquitoes is restricted: they move only when in *Blood Meal Seeking* or *Gravid* states (marked in red in Figure 1) to seek for resources. The resource-seeking process is modelled with random, non-directional flights with limited flight ability and perceptual ranges until they can perceive resources at close proximity, at which point, the flight becomes directional. At any point in the resource-seeking process, a mosquito’s neighbourhood is modelled as an eight-directional *Moore neighbourhood* (in cellular automata, a *Moore neighbourhood* comprises the eight cells surrounding a central cell on a two-dimensional square grid, or lattice). If the current cell and its neighbourhood do not contain any resource, the mosquito starts a random flight and moves randomly into one of the adjacent eight cells (like [10], the probability of moving into a diagonally-adjacent cell is set as half that of moving into a horizontally- or vertically-adjacent cell). If, on the other hand, it perceives a resource in the neighbourhood, it flies directly to the cell containing the resource.

**Figure 2:**
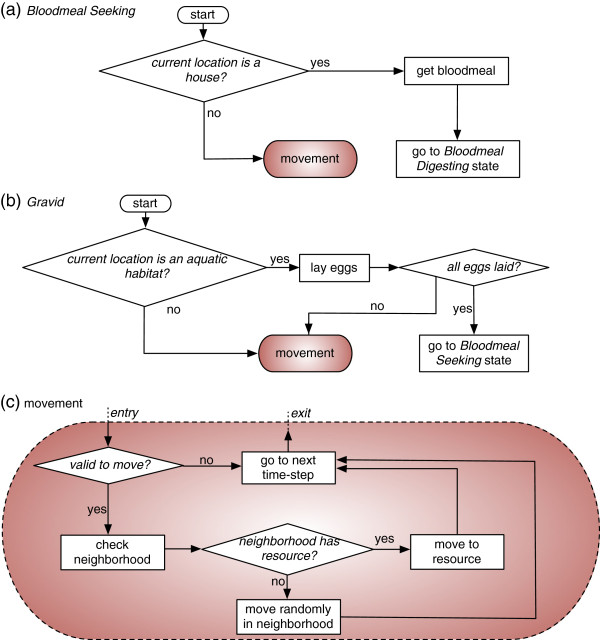
**Mosquito movement during resource seeking.** In the spatial ABM, female mosquitoes move in *Blood Meal Seeking* or *Gravid* states in order to seek resources (houses or aquatic habitats, respectively) in an eight-directional *Moore neighbourhood*. If a resource is perceived in the neighbourhood, the mosquito directly moves to it in the next time-step. Otherwise, it moves in random direction to an adjacent cell. In subfigure **(a)**, if the current location is not a house, the *Blood Meal Seeking* mosquito moves in order to find a house. In subfigure **(b)**, if the current location is not an aquatic habitat, the *Gravid* mosquito moves in order to find an aquatic habitat. In both cases, the mosquito would transition to subfigure **(c)**, which represents the detailed view of movement activities (that may occur in a single time-step) in subfigures **(a)** and **(b)**.

In the *Blood Meal Seeking* state, the mosquito looks for human houses, and the search continues until it successfully finds a house. In the *Gravid* state, the mosquito looks for an aquatic habitat, and once found, lays its eggs. The number of eggs it can lay is governed by the density-dependent oviposition rules (see [18,19] for details). If all of the eggs are laid, it goes to the *Blood Meal Seeking* state again, initiating a new gonotrophic cycle. Otherwise, it either remains in the same aquatic habitat or searches for another one to lay the remaining eggs, and this process continues until all the eggs are laid. The movement activities for both states are depicted as logical flowcharts in Figure 2.

### Model assumptions

The current work is theoretical. The presence of only one vector, *An. Gambiae*, is assumed. The vector life cycle dynamics is emphasized, and the parasite life cycle and the malaria transmission cycle are not yet included. Mosquitoes senesce, and their probability of death increases with age. The human population is modelled as static, i.e., humans do not move in space. All humans are assumed to be identical. For host-seeking, alternative hosts for blood-feeding (e.g., cattle) are not modelled, and the only blood meal-sources are humans in the houses. Daily temperature, variations in which can affect the model’s output (see [18,19] for details), is fixed at 25°C for this study. Seasonality and other weather/climate parameters are not included. Each aquatic habitat is set with a carrying capacity (*CC*) of 1,000 (see [18,20] for details). Time is modelled with hourly (instead of daily) time-steps, since this approach provides much more flexibility in modelling certain agent behaviours (e.g., host-seeking to start at a particular hour at night). For the grid-based landscapes, the size of each cell is set as 50 m, reflecting the limited perceptual range of *An. gambiae*[10]. For LSM, all aquatic habitats are treated indifferently, i.e., with no inherent differences in their attractiveness and productivity. For ITNs, the transient effects such as the decay of insecticide effectiveness of the bed nets are ignored. Complete usage (adherence) is assumed, i.e., humans provided with a bed net are always assumed to sleep under it during night. All ITN parameters (coverage, repellence and insecticidal effect) are assumed to be invariant over time, and any possible development of insecticide resistance in the mosquitoes is ignored.

Female adult mosquito abundance is treated as the primary output of the model. The associated *CC* of each aquatic habitat serves two purposes: 1) it limits the number of eggs a female mosquito may oviposit in an aquatic habitat (thus determining soft limits on larval density of the habitat); and 2) is used to model the Gravid female’s inclination to avoid less suitable (e.g., over-crowded) habitats. Unlike other studies [10,11], *CC* is not treated as a hard limit. When no intervention is in action, the mosquito population is governed by the combined carrying capacities of all aquatic habitats, and the density-dependent oviposition mechanism, which limits the potential number of eggs that a female mosquito may preferentially lay in an aquatic habitat, considering both the associated *CC* and the biomass already present in the habitat (for details, see [18,19]).

### Simulations

All simulations are started with 20,000 *Gravid* mosquitoes seeking human blood meals, which are initially placed at randomly selected houses. Each simulation is run 50 times and average results of all 50 runs are reported. Each simulation is run for at least one year. Intervention(s) are applied on day 100 and continued up to the end of the simulation. Thus, it is ensured that a long enough *warm-up* period has passed to reach a steady state (which, without any intervention, occurs around day 50), and that the results are reported after the simulation reaches equilibrium. Where applicable, percent reduction (PR) values in mosquito abundance are calculated by averaging 30-day abundances (after the population reaches steady state) from two intervals: *before* and *after* applying the intervention(s) to the base mosquito population. All simulations are submitted using the Sun Grid Engine (SGE) job scheduler, and run as single-threaded programs, in single-process per core mode in computing clusters with multiple cores.

### Applying LSM in isolation

To explore the impact of LSM in isolation (i.e., without any other intervention) and to replicate the results of GN-LSM, the 40 × 40 grid-based landscapes used in GN-LSM [10] are discretized and digitized. In the digitization process, the original tiny landscapes (from [10]) are enlarged, and gridlines are added to aid in measuring the objects’ coordinates. The coordinates are then measured by inspection. To locate the center of each object (an aquatic habitat or a house), distances (in both x- and y- axes) from the nearest gridlines are used. Whenever multiple objects overlap and appear to be rendered on top of one another, the center coordinates are inferred as best guesses (for example, in the original tiny landscapes (from [10]), if two objects seem to possess the same center coordinates, they are assigned to same or different cells in the digitized landscapes, depending on their distances from the nearest gridlines in the enlarged versions). The landscapes are then generated by using the landscape generator tool *VectorLand*. Each of the 18 landscapes, depicted in Additional file 5, contains 70 aquatic habitats (blue circles) and three different arrangements of 20 houses (black house icons): *diagonal*, *horizontal* and *vertical* (for details on these landscape patterns, see [10] and Additional file 5). For each arrangement, different LSM scenarios (targeted and non-targeted) are also constructed, as was done by [10]. The three targeted interventions (targeted removal of larval habitats) T1, T2 and T3 refer to the removal of aquatic habitats within 100, 200 and 300 m of surrounding houses, accounting for 4, 17 and 28 of 70 habitats, respectively. C1, C2 and C3 refer to non-targeted, random removal of the same numbers of aquatic habitats as the corresponding targeted interventions. Removal of an aquatic habitat makes it completely inaccessible to *Gravid* mosquitoes, and no eggs can be laid in it during oviposition. In practice, this is usually done by habitat modification (a category of LSM), which results in permanent change of land and water, and is performed by means that include landscaping, drainage of surface water, land reclamation and filling, coverage of large water storage containers, wells and other potential breeding sites, etc. [25]. Increasing LSM coverage, although affecting the larval population (by killing the biomass in the corresponding aquatic habitats), does not increase the mortality of adult mosquitoes; it just decreases the probability of successfully finding an aquatic habitat (and hence delaying the process) by adult females trying to oviposit. Note that the digitization of these landscapes from GN-LSM [10] (and later from GN-ITN [11]) is conducted primarily for validation, comparison and replication purposes. It is much easier and less time-consuming to generate new landscapes with any desired spatial distribution and parameter combinations using *VectorLand* (as shown later for applying LSM and ITNs in combination). However, to be able to directly compare the results with GN-LSM, and to adhere to the requirements of a standard replication process, the digitization of the original landscapes is necessary.

To compare the impact of LSM using the above landscapes, a fixed daily mortality rate (DMR) of 0.2 is used for the absorbing boundary in order to match the DMR of the GN-LSM study [10]. However, the original model uses age-dependent DMRs for some states in the life cycle of mosquito agents (age-dependent DMRs for all *Adult* states and the *Larva* state, and fixed DMRs of 0.1 for *Egg* and *Pupa* states) [18-20]. Hence, in simulations that use a non-absorbing boundary, age-dependent DMRs are used for all Adult states and the Larva state.

### Applying ITNs in isolation

Response of host-seeking mosquitoes to ITNs is modelled as a series of three ITN parameters: coverage C, repellence R and mortality M (note that the term *mortality* is used to refer to the insecticidal effect of the bed nets, i.e., the mortality concurred by ITNs). When a female mosquito (being in the *Blood Meal Seeking* state) finds a house, coverage is checked first to ensure whether the house is ITN-covered. If it is covered, repellence comes into action: the mosquito may be repelled by ITN and thus forced to search for another house. If it can avoid repellence, a random host is picked in the house. If the host sleeps under bed net, mortality comes into action: it may be killed due to mortality. If it survives the exposure to an ITN, depending on the ITN coverage scheme (see below), it either picks another random host in the same house or must search for another house. If, on the other hand, the host does not sleep under bed net, feeding is assumed to be always successful.

**Figure 3:**
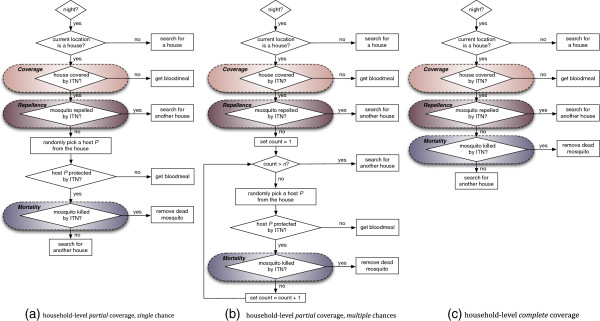
**Logical flowcharts for three different ITN coverage schemes.** The three different definitions (schemes) of ITN coverage depicting the response of an individual female mosquito to ITNs: **(a)** household-level *partial* coverage with *single* chance, **(b)** household-level *partial* coverage with *multiple* chances and **(c)** household-level *complete* coverage. In subfigure **(b)**, *n* denotes the number of persons in the house. For descriptions, see Methods.

As stated before, simulating the three different definitions (schemes) of ITN coverage is important because although different studies used different schemes [2,8,11,12,30], none (including ABMs and mathematical models of malaria) actually compared their relative impacts side-by-side. Without a precise definition of the scheme used in a particular model, the task of replication becomes much harder. Hence, the comparison of results from using the three schemes may guide future modellers to decide and choose from which one to use in their models. These three schemes of ITN coverage differ by the number of persons actually covered by bed nets in a house that is under ITN coverage. Note that the same household-level coverage in different schemes may yield different population-level coverage, as shown in Table 2. These three schemes are depicted as logical flowcharts in Figure 3. Household-level coverage and population-level coverage are defined as:

$$\begin{array}{l} \text{Household}‒\text{level coverage}\;\left(\%\right)\\ \;=(\text{Number of houses with coverage}/\\ \;\text{Total number of houses})\times 100 \end{array}$$

$$\begin{array}{l} \text{Population}‒\text{level coverage}\;\left(\%\right)\\ \;=(\text{Number of bed net users}/\\ \;\text{Total human population})\times 100 \end{array}$$

**Table 2:** Population profiles for varying levels of ITN coverage with multiple coverage schemes

**ITN coverage**	**Partial coverage scheme**			**Complete coverage scheme**		
	**Number of bed net users**	**Number of non-users**	**Population-level coverage (%)**	**Number of bed net users**	**Number of non-users**	**Population-level coverage (%)**
0.4	40	145	21.62	76	109	41.08
0.6	60	125	32.43	110	75	59.46
0.8	80	105	43.24	153	32	82.70
1.0	100	85	54.05	185	0	100

The table shows the differences in household-level coverage and population-level coverage, as well as the variation in number of bed net users and non-users, for varying levels of ITN coverage with multiple coverage schemes (see Methods and Figure 3). To match GN-ITN [11], the human population is set as 185. In the partial scheme, only two persons are protected by a bed net in a ITN-covered house. On the other hand, in the complete scheme, all persons in the house are protected by bed nets in a ITN-covered house. For example, with 60% ITN coverage, in the partial scheme, 60 persons are protected by bed nets, yielding a population-level coverage of 32.43% (60 / 185 × 100); with the same ITN coverage, in the complete scheme, 110 persons are protected by bed nets, yielding a population-level coverage of 59.46% (110 / 185 × 100).

The distinction between partial and complete schemes becomes apparent when the respective numbers for varying levels of ITN coverage are compared. As shown in Table 2, for any ITN coverage level (column 1), the complete coverage scheme has almost *twice* the number of bed net users (compare columns 2 and 5) and the population-level coverage (compare columns 4 and 7) than those in the partial coverage scheme.

In household-level *partial* coverage with *single* chance for host-seeking, each house with ITN coverage is assigned a single bed net, and two randomly selected persons are protected by the bed net (irrespective of the total number of persons in the house). Once a host-seeking mosquito enters a ITN-covered house, and is not deterred by the repellence, it gets a single chance of obtaining a blood meal by picking a random host in the house. Since at most two persons can sleep under the bed net, the probability of a random host sleeping under the bed net is 2/*n*, where *n* is the number of persons in the house. Thus, the probability to obtain a blood meal from a non-protected host in the house is 1 - 2/*n*. If the host is protected (sleeps under the bed net), the mosquito cannot get a blood meal but still runs the risk of being killed by the ITN mortality (insecticidal effect of the bed nets). If it can survive, it must start searching for another house. Otherwise (i.e., if the host is unprotected), the mosquito gets a blood meal.

The second scheme, household-level *partial* coverage with *multiple* chances for host-seeking, works similarly as the first one, except for the fact that a host-seeking mosquito gets *n* chances in the same house (where *n* is the number of persons in the house). If it cannot get a blood meal within *n* chances and still survives the ITN mortality, it must start searching for another house. Note that with this scheme, even though the mosquito gets multiple chances for host-seeking, it also encounters the risk of being exposed to the ITN mortality each time (if the randomly selected host sleeps under bed net). With both these schemes, even when all houses are ITN-covered (i.e., 100% household-level coverage), a portion of the human population may still remain unprotected, and thus, the vector population may not be completely suppressed. With the last scheme, household-level *complete* coverage, if a house is ITN-covered, all persons in the house are protected by bed nets (and hence the term *complete* is used). This can simulate, for example, an ITN study over a region where there are enough bed nets to protect every person in a ITN-covered house. In this scheme, when a host-seeking mosquito enters a ITN-covered house and is not deterred by the repellence, it cannot get a blood meal (because all persons are covered), and must search for another house. Thus, it incurs additional delays and risks for the mosquito to be eventually successful in obtaining a blood meal.

To evaluate the impact of ITNs, the settings used in the GN-ITN study [11] are replicated. Using *VectorLand*, the single 40 × 40 grid-based landscape used in GN-ITN is digitized by the same procedure as described before (to digitize the 18 landscapes from GN-LSM). The landscape, depicted in Additional file 6, contains 90 aquatic habitats (blue circles) that are randomly distributed, and 50 houses (black squares) that are arranged diagonally. To run the simulations, representative sample values are used from the parameter space for the three ITN parameters (coverage, repellence and mortality). The parameter values used are shown in Table 3. Combining these values yields 60 distinct parameter combinations. For each combination, 50 replicated simulations are run for each of the three coverage schemes (see description above and Figure 3), and the average results are reported. A non-absorbing boundary is used for all cases.

**Table 3:** Parameter space for applying ITNs (in isolation)

**Parameter**	**Values**
Coverage (C)	0.4, 0.6, 0.8, 1.0
Repellence (R)	0.2, 0.5, 0.9
Mortality (M)	0.0, 0.25, 0.5, 0.75, 1.0

Representative sample values for the three ITN parameters (coverage, repellence and mortality) are used to evaluate the impact of ITNs in isolation. For each of the three ITN coverage schemes (see Methods and Figure 3) and 60 distinct parameter combinations, 50 replicated simulations are run, yielding a total of 15,000 simulations. Results are shown in Figures 8, 9 and Additional files 9, 10, 11 and 12.

### Population profiles for varying levels of ITN coverage with different schemes

The 50 houses in the landscape used in the GN-ITN study [11] accommodate a total human population of 185, with the average of household residents being 3.7 (with standard deviation of 1.2). The same distribution is used to generate the population profiles, ensuring that the total human population is 185, with each house having at least two residents.

### Applying LSM and ITNs in combination

**Figure 4:**
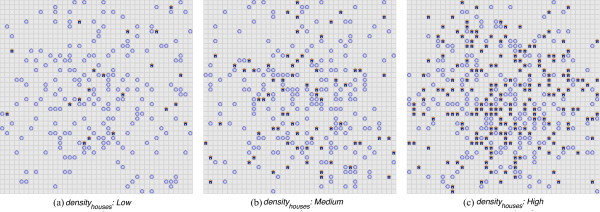
**Sample landscapes used for applying LSM and ITNs in combination.** Subfigures **(a)**, **(b)** and **(c)** represent three 40 × 40 landscapes, each containing 200 aquatic habitats, with different densities of houses (*density*_*houses*_): **(a)***Low* (20), **(b)***Medium* (70) and **(c)***High* (200), respectively, with corresponding human population densities of 100, 350 and 1,000, respectively. Aquatic habitats and blood meal locations are shown as blue circles and house-shaped icons, respectively. For 240 distinct parameter combinations (involving *density*_*houses*_, LSM coverage, ITN coverage and ITN mortality, as shown in Table 4), similar landscapes are generated and 50 replicated simulations are run for each (see Figure 11 for results). All landscapes are generated using the landscape generator tool *VectorLand*.

To evaluate the impact of applying LSM and ITNs in combination, three 40 × 40 landscapes are created (using *VectorLand*), with varying densities of houses (blood meal locations), *density*_*houses*_, where the density refers to the number of houses: *Low* (20), *Medium* (70) and *High* (200). For each *density*_*houses*_, a corresponding human population density (total human population) is also set: *Low* (100), *Medium* (350) and *High* (1,000). Sample landscapes with the three *density*_*houses*_ levels are shown in Figure 4. Parameter values used to run simulations with these three landscapes are shown in Table 4. For each of the 240 distinct parameter combinations, 50 replicated simulations are run, yielding a total of 12,000 simulations. For all cases, the household-level complete coverage scheme is used for ITNs, and ITN repellence (R) is ignored (i.e., R is set to 0.0). Initially, aquatic habitat density is fixed at 50 per km^2^ (in 40 × 40 landscapes, since each cell represents 50 m × 50 m, the 200 aquatic habitats are scattered across an area of 4,000,000 m^2^, or 4 km^2^), and later reduced as LSM coverage is increased.

**Table 4:** Parameter space for applying LSM and ITNs

**Parameter**	**Values**
*density*_*houses*_	*Low* (20), *Medium* (70), *High* (200)
Population density	*Low* (100), *Medium* (350), *High* (1,000)
LSM coverage	0.0, 0.1, 0.3, 0.6, 0.9
ITN coverage	0.0, 0.25, 0.5, 1.0
ITN mortality	0.0, 0.3, 0.7, 1.0

Representative sample values used to evaluate the impact of applying LSM and ITNs in combination. For 240 distinct parameter combinations, 50 replicated simulations are run, yielding a total of 12,000 simulations. For ITNs, the household-level complete coverage scheme is used (see Methods), and repellence is ignored. Within a 40 × 40 landscape, *density*_*houses*_ refers to the number of houses (blood meal locations), and population density refers to the total human population (for the corresponding *density*_*houses*_). For all cases, initially, aquatic habitat density is fixed at 200, and later reduced as LSM coverage is increased. Results are shown in Figure 11 and Additional file 13.

## Results

### Impact of LSM (in isolation)

**Figure 5:**
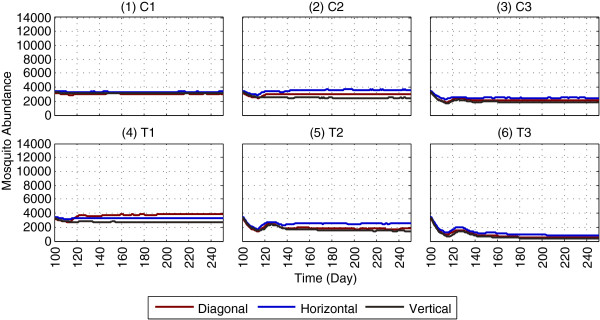
**Impact of LSM on mosquito abundance, using an absorbing boundary.** The figure depicts the results of applying LSM (in isolation), as the results of GN-LSM [10] are replicated along with the scenarios and landscapes. Each subfigure represents a specific LSM scenario. Subfigures **(1)**-**(3)**, denoted as C1, C2 and C3, refer to the non-targeted, random removal of aquatic habitats. Subfigures **(4)**-**(6)**, denoted as T1, T2 and T3, refer to the targeted removal of aquatic habitats within 100, 200 and 300 m of surrounding houses, respectively. The non-targeted scenarios remove the same numbers of aquatic habitats as in the corresponding targeted scenarios (for example, both C1 and T1 remove 4 habitats). Within each subfigure, the Diagonal, Horizontal and Vertical plots represent abundances (for the specified LSM scenario) for three different arrangements of houses in the landscapes (see Additional file 5 for the landscapes). With an absorbing boundary, mosquitoes are killed when they hit an edge of the landscape’s boundary. The x-axis denotes simulation time (in days) and the y-axis denotes mosquito abundance. For brevity, the 150 days results (day 100 - day 249) are shown; the full one-year results are given in Additional file 7. This figure represents averages of a total of 900 (18 × 50) simulations.

**Figure 6:**
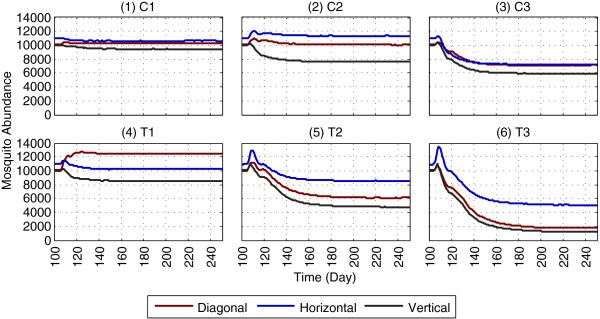
**Impact of LSM on mosquito abundance, using a non-absorbing boundary.** The figure depicts the results of applying LSM (in isolation), as the results of GN-LSM [10] are replicated using a non-absorbing boundary. With a *non-absorbing boundary*, when mosquitoes hit an edge of the landscape’s boundary, they enter the landscape from the edge directly opposite of the exiting edge (and thus are not killed due to hitting the edge). Subfigures **(1)**-**(3)**, denoted as C1, C2 and C3, refer to the non-targeted, random removal of aquatic habitats. Subfigures **(4)**-**(6)**, denoted as T1, T2 and T3, refer to the targeted removal of aquatic habitats within 100, 200 and 300 m of surrounding houses. Results within each subfigure are obtained using the same parameters as in the corresponding subfigure of Figure 5 (except the boundary type). For brevity, the 150 days results (day 100 - day 249) are shown; the full one-year results are given in Additional file 8. This figure represents averages of a total of 900 (18 × 50) simulations.

Impact of applying LSM in isolation on mosquito abundance is shown in Figures 5 and 6, using absorbing and non-absorbing boundaries, respectively (the reproduced landscapes used for LSM application are shown in Additional file 5). For brevity, the 150 days results (day 100 - day 249) are shown, with LSM being applied on day 100; the full one-year results are shown in Additional files 7 and 8. To compare the results with GN-LSM [10], the PR values in abundances are calculated, which are shown in Table 5.

**Table 5:** **Percent reductions in abundance with LSM (applied in isolation): a comparison with GN-LSM [**[10]**]**

		**C1**	**T1**	**C2**	**T2**	**C3**	**T3**	**Reference**
*Diagonal*	GN-LSM (Absorbing)	4.2	38.4	8	100	69.6	100	[10]
	Absorbing	2.08	−21.63	3.56	43.82	31.74	85.32	This study
	Non-absorbing	−1.82	−23.24	0.22	39.55	29.72	82.65	This study
*Horizontal*	GN-LSM (Absorbing)	8.9	−5.7	44	100	34.3	100	[10]
	Absorbing	4.35	7.37	−3.96	29.03	29.3	78.82	This study
	Non-absorbing	3.25	6.71	−3.27	22.29	34.16	54.01	This study
*Vertical*	GN-LSM (Absorbing)	2.8	30.6	16.67	100	33.14	100	[10]
	Absorbing	5.21	15.45	24.17	55.54	43.21	91.79	This study
	Non-absorbing	5.32	14.32	23	52.13	40.45	88.20	This study

These results are obtained using LSM only (without ITNs). Rows labelled with Diagonal, Horizontal and Vertical refer to the different arrangements of houses in the landscapes (see Methods and Additional file 5). C1, C2, C3 and T1, T2, T3 refer to non-targeted and targeted removal scenarios, respectively. Each value (in the rows labelled as Absorbing and Non-absorbing) represents the average percent reduction of 50 simulation runs.

Although the two models (i.e., the GN-ABM [10] and the ABM used in this study) differ in several assumptions, in most cases, general agreement in changes in PRs (i.e., an increase or decrease) is observed for the different landscapes, as shown in Figures 5, 6 and Table 5. For all landscape types (*Diagonal*, *Horizontal* and *Vertical*), in the model, the absorbing boundary almost always (in 17 out of 18 scenarios, i.e., 94% cases) yields larger PR than that of the non-absorbing case within the same scenario. While this trend is generally expected due to the additional (but unrealistic) killing effect of the absorbing boundary, this indicates the validity of results obtained from comparing the models using different boundary types.

It is interesting to observe that in Figures 5 and 6, except for scenario T1, abundances in all other scenarios for the *Horizontal* landscape are greater than those for the *Diagonal* and *Vertical* landscapes. This is because the average distance between aquatic habitats and blood meal locations (when both of these resource types are ranked according to distances from one another) for the *Horizontal* landscape is less than those for the *Diagonal* and *Vertical* landscapes. As a result, female mosquitoes need to travel shorter average distances in the *Horizontal* case in order to find resources, and thus completing their gonotrophic cycles. For scenario T1 (which is obtained by removing four aquatic habitats from the baseline landscapes), however, abundance for the *Diagonal* landscape is greater than that for the *Horizontal* landscape. To explore why, the *effective shortest distances* (*ESDs*) between each of the four removed aquatic habitats, and to their seven nearest blood meal locations, are measured. *ESD* measures the shortest distance, in units of number of cells, between the source and the destination cells (recall that each cell in the landscape is 50 m × 50 m; thus, x *ESD* means x × 50 m), and includes diagonal paths wherever necessary, since mosquitoes are allowed to move diagonally in the ABM. It turns out that *ESD*_*Diagonal*_ = 143 and *ESD*_*Horizontal*_ = 197, i.e., *ESD*_*Diagonal*_ <*ESD*_*Horizontal*_ (see Additional file 5 for the specific landscapes). This suggests that removal of these four aquatic habitats in scenario T1 has less impact for the *Diagonal* landscape than for the *Horizontal* landscape - female mosquitoes can find blood meals more easily by travelling less distances in the former (*Diagonal*) case, resulting greater abundances.

### Impact of single *vs* multiple simulation runs

**Figure 7:**
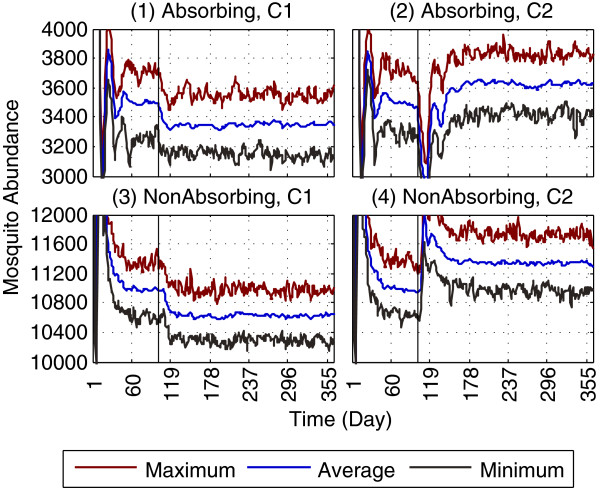
**Importance of performing multiple simulation runs.** The importance of performing multiple simulation runs (instead of a single run) can be seen by comparing abundances for maximum, minimum and average cases. Four sample scenarios are shown. Subfigures **(1)** and **(2)** refer to the non-targeted, random removal of aquatic habitats (scenarios C1 and C2, respectively, from Figures 5-6) using an *absorbing* boundary. Subfigures **(3)** and **(4)** refer to the same scenarios, using a *non-absorbing* boundary. For each scenario, the results are derived from 50 replicated runs. The maximum, minimum and average represent the maximum, minimum and average abundance values obtained across all 50 replicated runs in each time-step, respectively. The average case is used in all simulations reported in this study. Note that the scales along y-axes of the subfigures are purposefully modified (zoomed in) to highlight the actual differences between the three cases. See Additional file 5 for the landscapes.

As explained before, different simulation runs (with identical parameter settings) can produce significantly different results due to the stochasticity involved while generating random draws from the probability distributions. The importance of multiple simulation runs, instead of a single run, is depicted in Figure 7, where the *maximum*, *minimum* and *average* abundance values, obtained in each time-step across 50 replicated runs from four sample scenarios, are derived. As evident from Figure 7, the *average* plot lies within a band (envelope) defined by the *maximum* and *minimum* plots. If replication is not done (by performing multiple simulation runs), the results could have potentially taken any trajectory bounded within the band, and thus would have been less reliable. Also, note that the *average* plot is much smoother than the other two, suggesting much less abrupt changes (caused by the random events). All simulation results reported in this work represent the same replication mechanism of multiple runs.

### Impact of boundary types

As stated above, in 94% cases, use of an absorbing boundary yields less abundance than that with a non-absorbing boundary. Also, with an absorbing boundary, even before applying LSM (i.e., before day 100), abundances with all landscapes are too low when compared to those with a non-absorbing boundary (see Additional files 7 and 8 for the full one-year results). Since at the beginning of all simulations, female mosquitoes start their activities from randomly selected houses, a good portion of them aggregate around these clumped houses. In many cases, these clumped houses have comparatively far smaller average distance to their nearest edges in the landscape (see Additional file 5 for the landscapes). As a result, female mosquitoes that start moving around from these houses find an edge much quicker (and thus being killed) than those which start from other houses. Thus, just due to using an absorbing boundary, more mosquitoes die out due to the additional unrealistic killing effect imposed by the absorbing boundary. This suggests the importance of using a non-absorbing boundary in the ABM to avoid the potential bias created by a specific boundary type.

### Impact of ITNs (in isolation)

**Figure 8:**
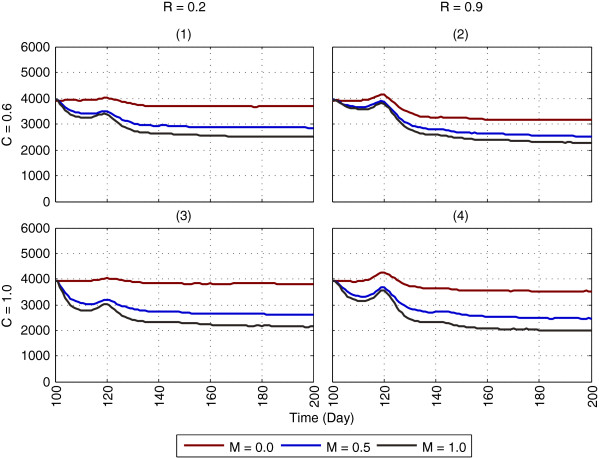
**Impact of ITNs on mosquito abundance, using household-level partial coverage with multiple chances for host-seeking.** The figure depicts the results of applying ITNs (in isolation) with household-level partial coverage, as the results of GN-ITN [11] are replicated. Each subfigure represents a specific combination of coverage (C) and repellence (R) for ITNs: **(1)** C = 0.6, R = 0.2, **(2)** C = 0.6, R = 0.9, **(3)** C = 1.0, R = 0.2 and **(4)** C = 1.0, R = 0.9. Within each subfigure, each colour-coded plot represents a specific mortality (M) value for ITNs (e.g., M = 0.5), with mortality (M) colour keys at the bottom of the figure. The x-axis denotes simulation time (in days) and the y-axis denotes mosquito abundance. For brevity, the 100 days results (day 100 - day 199) for a subset of the parameters (from Table 3) are shown; the full one-year results, for the entire parameter space, for partial coverage schemes with single and multiple chances, are shown in Additional files 9 and 10, respectively. The figure represents averages of a total of 600 (2 × 2 × 3 × 50) simulations. A *non-absorbing boundary* is used. For partial coverage schemes, see Figure 3 and Methods.

**Figure 9:**
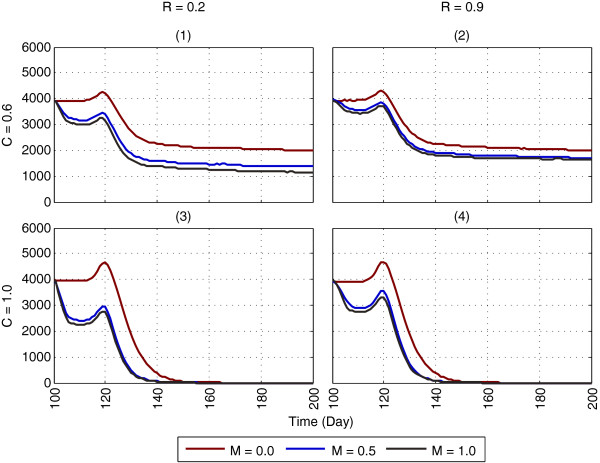
**Impact of ITNs on mosquito abundance, using household-level complete coverage.** The figure depicts the results of applying ITNs (in isolation) with complete coverage, as the results of GN-ITN [11] are replicated. Each subfigure represents a specific combination of coverage (C) and repellence (R) for ITNs: **(1)** C = 0.6, R = 0.2, **(2)** C = 0.6, R = 0.9, **(3)** C = 1.0, R = 0.2 and **(4)** C = 1.0, R = 0.9. Within each subfigure, each colour-coded plot represents a specific mortality (M) value for ITNs (e.g., M = 0.5), with mortality (M) colour keys at the bottom of the figure. For brevity, the 100 days results (day 100 - day 199) for a subset of the parameters (from Table 3) are shown; the full one-year results, for the entire parameter space, are shown in Additional file 11. The figure represents averages of a total of 600 (2 × 2 × 3 × 50) simulations. A non-absorbing boundary is used. For other details, see legend of Figure 8. For the complete coverage scheme, see Figure 3c and Methods.

**Figure 10:**
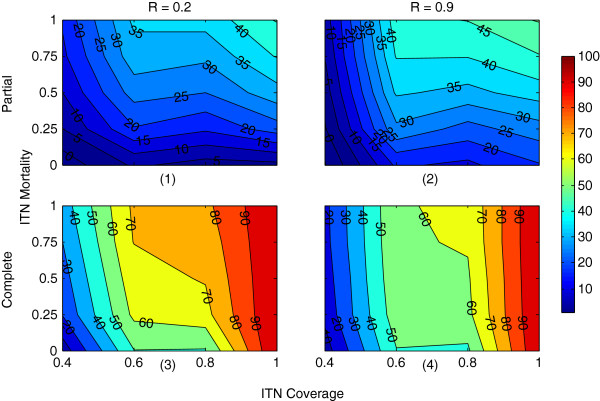
**Percent reductions in mosquito abundance by ITNs, applied in isolation, comparing household-level partial coverage and complete coverage.** The x-axis denotes ITN coverage and the y-axis denotes ITN mortality. Each subfigure represents a specific combination of coverage scheme (household-level *partial* coverage or household-level *complete* coverage) and repellence (R) for ITNs: **(1)** the partial scheme, R = 0.2, **(2)** the partial scheme, R = 0.9, **(3)** the complete scheme, R = 0.2 and **(4)** the complete scheme, R = 0.9. ITN is applied at day 100 in the 40 × 40 grid-based landscape (see Additional file 6) with 50 houses having a total human population of 185. The percent reduction (PR) values, represented as filled contour plots in each subfigure, are calculated from data used in Figures 8 and 9. The colourbar on the right quantifies the PR isolines. The figure depicts selected results that involve a subset of the parameters from Table 3; results for the entire parameter space (see Table 3) are depicted in Additional file 12.

Impact of ITNs in isolation on mosquito abundance is shown in Figures 8 and 9, using household-level *partial* coverage (with multiple chances for host-seeking) and *complete* coverage, respectively (the reproduced landscape from GN-ITN [11] used for ITN application is shown in Additional file 6). For brevity, the 100 days results (day 100 - day 199), involving a subset of the parameters (from Table 3) are shown, with ITNs being applied on day 100. The full one-year results for the entire parameter space are shown in Additional files 9, 10 and 11. Figure 10 and Additional file 12 show PR values in abundance obtained by applying ITNs for household-level partial coverage (with multiple chances) and complete coverage for host-seeking.

The two partial coverage schemes with *single* or *multiple* chances produce little difference when compared (see Additional files 9 and 10). Searching for a host within the same house for n times does not give much leverage to the mosquito, because each time, if the randomly-picked host is protected by a bed net, the risk of being exposed to the insecticidal effect of ITNs (and thus getting killed) still exists. Since GN-ITN [11] is based on partial coverage, their abundance results are compared with Figure 8 (which shows household-level partial coverage with multiple chances for host-seeking). As coverage C increases, abundance is eventually reduced from 4,000 to ≈ 2,000, as shown in Figure 8. This seems more plausible as opposed to achieving a 100% reduction in abundance as was shown by GN-ITN [11], because with the partial coverage scheme, since only 54% of the human population are protected by bed nets, a portion of the mosquitoes can still find enough blood meals, and hence a complete suppression of the mosquito population cannot be expected.

As shown in Figure 9 and Additional files 11 and 12, with household-level complete coverage scheme, abundance is reduced from 4,000 to ≈ 1,000 when coverage is in the range 60% < C ≤ 80% and repellence R is not too high (see subfigure 1 in Figure 9). As C approaches 100% (i.e., all humans are protected by bed nets), irrespective of repellence, abundance can be completely suppressed, as seen in subfigures 3 and 4 in Figure 9. However, too high repellence (e.g., 0.5% ≤ R ≤ 0.9), though unlikely to be present in commonly used insecticides in real-world scenarios, can have a detrimental effect on vector control (by increasing abundance) with the same levels of coverage and mortality, but the degree of this negative impact is reduced as coverage increases (see subfigure 2 in Figure 9, subfigure 4 in Figure 10, and subfigures 7-8 in Additional file 12). As seen in subfigures 5-7 in Additional file 12, when R ≤ 0.5, around 60% PR can be achieved with coverage and mortality being as low as ≈ 60% and ≈ 30%, respectively. However, when 0.5 < R ≤ 0.9, to achieve the same PR, the coverage needs to be as high as ≈ 85%. Also, R = 0.9 means 90% of the host-seeking mosquitoes are driven away from the house before the ITN mortality can play any role (see the complete coverage flowchart in Figure 3c). This is why mortality seems to have less impact in subfigure 4 than in subfigure 3 in Figure 10.

Interestingly, with the complete coverage scheme, even with no ITN mortality, very high PR (around 80%) can be achieved with high coverage (≈ 90%), irrespective of repellence (see subfigures 3-4 in Figure 10). With 90% coverage, around 90% of the population sleeps under bed nets. Since the ABM assumes complete usage of bed nets, and the *An. gambiae* mosquitoes are almost exclusively anthropophilic and highly endophagic, no blood meal can be obtained from sources other than humans, or during daytime. Thus, though no mosquitoes are killed due to ITN mortality, they cannot complete their gonotrophic cycles (because ≈ 90% of the host-seeking attempts fail), and eventually, the mosquito population dies out.

### Impact of combining LSM and ITNs

**Figure 11:**
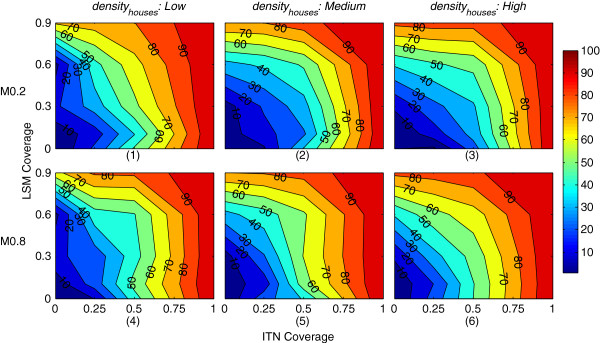
**Percent reductions in mosquito abundance as a function of LSM coverage and ITN coverage when LSM and ITNs are applied in combination.** The x-axis denotes ITN coverage and the y-axis denotes LSM coverage. Each subfigure represents a specific combination of density of houses (*density*_*houses*_) and mortality (M) for ITNs: subfigures **(1)**-**(3)** represent M = 0.2 with *density*_*houses*_ of *Low*, *Medium* and *High*, respectively; subfigures **(4)**-**(6)** represent M = 0.8 with *density*_*houses*_ of *Low*, *Medium* and *High*, respectively. ITN repellence (R) is fixed at 0.5. Each simulation is run for one year; both LSM and ITNs are applied at day 100, and continued up to the end of the simulation. Each subfigure represents filled contour plots where the isolines are labelled with specific percent reduction (PR) values. The colourbar on the right quantifies the PR isolines. The figure represents average percent reduction values of a total of 6,000 (3 × 5 × 4 × 2 × 50) simulations. For ITNs, household-level complete coverage scheme is used (see Figure 3c). A *non-absorbing boundary* is used. Sample landscapes with the three *density*_*houses*_ levels are shown in Figure 4. The figure depicts selected results that involve a subset of the parameters from Table 4; results for the entire parameter space are depicted in Additional file 13.

To evaluate the impact of applying LSM and ITNs in combination, three 40 × 40 landscapes are used with varying *density*_*houses*_ (see Methods). The results, depicted as PR values in mosquito abundance, are shown in Figure 11 and Additional file 13 (PRs are calculated as described before). Figure 11 depicts selected results that involve a subset of the parameters from Table 4; Additional file 13 depicts results that involve the entire parameter space. In these figures, each subfigure represents a filled contour plot where the isolines are labelled with specific PRs, whose magnitudes are shown in the colourbar on the right. Each row represents a specific mortality (M) value for ITNs (e.g., M = 0.2), as marked on the left. Each column represents a specific *density*_*houses*_, as marked on the top.

Figure 11 indicates some interesting observations. First, impact of ITN mortality (M) becomes increasingly important as the *density*_*houses*_ increases. Comparing the subfigures column-wise, increasing ITN mortality (M) has less impact on the landscape with *Low density*_*houses*_ than with *Medium* or *High* cases. With *Low density*_*houses*_, number of available human hosts is also low, making the number of host-seeking events much lower than the other two cases. Less host-seeking events in turn mean reduced possibility of a mosquito being in contact with an ITN, and as a result, increasing ITN mortality (M) cannot affect PR values as greatly as it can with the other two cases. In general, as ITN mortality and *density*_*houses*_ increase, more successes with the combined interventions are observed, indicating the importance of at least some ITN mortality being there when ITN is applied.

Increase in ITN mortality (M) also influences the general shape of the PR isolines. With *High density*_*houses*_ (column 3), as M increases, the combined interventions become more effective, as seen by the increases of higher PR values: for PR > 40%, the corresponding area increases from 70.83% (for M = 0.2) to 83.33% (for M = 0.8). This trend is also seen for *Low* and *Medium* densities (columns 1 and 2), with the *Low* density column having the least impact.

Next, considering the impact of each intervention in isolation (i.e., looking exactly at both the x-axis and y-axis PR values with y = 0 and x = 0, respectively) in Figure 11, on a per-row or per-column basis, the rate of changes in PR is similar across all subfigures. For example, with *Low density*_*houses*_ at subfigures 1 and 4 (column 1), looking at the y-axis (i.e., at x = 0, meaning when ITN is ignored, and LSM coverage is gradually increased), the isolines of PR values intersect the y-axes at approximately similar intervals (e.g., *PR*_*10*_ with LSM coverage ≈ 0.18, *PR*_*20*_ with LSM coverage ≈ 0.63, etc.). Similar trends are observed for columns 2 and 3, and also across the x-axis (i.e., at y = 0, meaning when LSM is ignored, and ITN coverage is gradually increased). This ensures that without the presence of the other intervention, both LSM and ITNs, with their respective parameters varied, yield significant impact on abundance, and confirms the first notion discussed before (ensuring the individual efficacy of each intervention).

Next, when ITN Mortality (M) is non-zero (i.e., the bed nets are at least partially lethal to mosquitoes), increasing ITN Coverage is more effective in reducing mosquito abundance (i.e., increasing PR) than increasing LSM Coverage, which is observed by the more pronounced increase in PR across the x-axis than up the y-axis. This observation is in agreement with similar results obtained in reducing the basic reproductive number of malaria (*R*_*0*_) by Yakob and Yan [2]. However, as seen in row 1 of Additional file 13, with non-lethal ITNs (M = 0), the efficacies of both interventions approach more equivalency as the *density*_*houses*_ approaches from *Low* to *High*. Also, comparing the subfigures column-wise in Figure 11, integrating both interventions yield more synergistic effect as the *density*_*houses*_ approaches from *Low* to *High*. Again, this trend agrees with similar results obtained in reducing *R*_*0*_ in [2].

Not surprisingly, the increases in PR values indicate more synergistic patterns when all parameters are in effect (i.e., have non-zero values). For example, looking at subfigures 2, 3, 5 and 6, where the *density*_*houses*_ is *Medium* to *High*, and M is in the range 0.2-0.8, increasing coverages of both interventions yield more synergistic benefits, as indicated by the more convexity of the PR isolines in general. In these cases, with sufficient number of host-seeking events, and ITNs in action with some mortality (insecticidal effect), both interventions play more effective roles in reducing abundances, and thus increasing PRs.

## Discussion

In general, with LSM applied in isolation, the replicated results agree with the major findings by GN-LSM [10] that LSM coverage of 300 m surrounding all houses can lead to significant reductions in abundance, and, while targeting aquatic habitats to apply LSM, distance to the nearest houses can be an important measure. However, as shown by the model, some of the underlying assumptions in the GN-LSM model could have seriously affected their predicted outcomes. To be specific, reporting results from a single simulation run and the use of an absorbing boundary could lead to substantially different results, invalidating the findings and thereby diminishing the predictive power of the models. Also, without a more sophisticated spatial metric that can capture the interrelations of different resources in different landscapes, simplistic features such as the general arrangement pattern of houses (e.g., diagonal, horizontal and vertical) are insufficient to capture a landscape’s potential to transmit the disease. For example, comparing the most restrictive cases (T3) of LSM application, the reduction in abundance is more prominent with a non-absorbing boundary (from ≈ 10,000 to ≈ 1,800, as shown in subfigure of Figure 6) than with an absorbing boundary (from ≈ 3,000 to ≈ 500, as shown in subfigure 6 of Figure 5). Due to the random distributions of houses and aquatic habitats in the three selected patterns, the reduction effects remain unpredictable, depending on factors such as the proximity of the resources to the boundaries of the landscapes. When applied to different (e.g., more general or specific) conditions, these assumptions may produce misleading results. The modified assumptions, as implemented in this study, provide new insights, and potentially more accurate results under certain conditions.

It is implausible to expect 100% reductions in abundance even with the most restrictive application of LSM (T3 in Figures 5, 6 and Table 5). This is because even with an absorbing boundary, some mosquitoes would always survive by roaming around in different parts of the landscape, instead of hitting the edges of the boundary (and hence dying out). This is observed in the results - the highest PR value obtained is 91.79% with scenario T3 using an absorbing boundary, as opposed to 100% observed in several cases in the GN-LSM study [10].

In few cases, negative PR values are obtained (see Table 5), suggesting that the abundances actually increase after applying LSM. A closer look at the landscapes (see Additional file 5) reveals that these cases are associated with the removal of a small fraction of all aquatic habitats (4 out of 90 for C1 and T1) by LSM. Recall that in the ABM, abundance is governed by the *CC* of aquatic habitats and the density-dependent oviposition mechanism. Removal of only a few nearby habitats may actually save a mosquito from wasting its time trying to search, locate, and compete in laying eggs in the already-crowded habitats, and instead be more productive by finding comparatively less-crowded habitats which are within close vicinity.

This points to an important insight: if the mosquito population in the environment is not unrestricted (i.e., it is restricted to be within the limit of the environment’s overall capacity, as in the ABM), and some stages of the mosquito biology are governed by special mechanisms (e.g., density-dependent oviposition), then removal of only an insufficient number of aquatic habitats may, in some cases, increase the abundance. Thus, before actually applying LSM, it may be crucial to estimate its impact (to achieve the desired level of success) by simulating varying levels of coverage.

As expected, with ITNs, different definitions of ITN coverage can lead to significantly different results. The household-level partial coverage schemes can provide only ≈ 50% reduction in abundance with 100% coverage and 100% mortality. This means that even when each house is equipped with one bed net (which, overall, covers only ≈ 54% of the human population), this scheme cannot perform even anywhere close to suppress abundance. On the other hand, the household-level complete coverage scheme can provide as much as 70% reductions in abundance with ≥ 85% coverage and mortality as low as 25%. With this scheme, when the coverage is 100%, abundance can be completely suppressed even when no mortality is in action (i.e., M = 0.0), as shown in subfigures 3-4 in Figure 9. This is expected: since every person in every house is protected by bed nets, the host-seeking mosquitoes cannot find unprotected hosts to obtain blood meals. While modelling the impact of ITNs, these distinctions should be clearly marked, and the choice of the ITN coverage scheme should be made carefully.

In general, repellence, which drives the host-seeking mosquito away from a house, can have a detrimental effect on vector control when the risk (additional delay in search etc.) of finding an unprotected host in another house is less than that in the same house. With the complete coverage scheme, since every person in the house (with ITN coverage) is protected by bed net, the above turns out to be true. However, as coverage C increases, more houses fall within the range of coverage, and the probability of finding an unprotected host (in another house) during the next search decreases. Thus, with increasing coverage, the negative impact incurred by too high repellence gets reduced, as evident in the first three rows (subfigures 1-9) of Additional file 11.

On the other hand, with household-level partial coverage schemes (both with single or multiple chances), this effect is almost absent (see Figure 8, subfigures 1-2 of Figure 10, and Additional files 9, 10 and 12). Recall that with partial coverage schemes, every person in the same house (with ITN coverage) may not be protected by a bed net. Thus, the mosquito may find an unprotected host in the same house. If it is repelled too often (due to high repellence), it is being deprived of its current positional advantage, and the risk of finding an unprotected host in another house may not be well-justified.

Interestingly, the use of a specific boundary type does not have significant impact for this particular landscape (see Additional file 6). Using absorbing and non-absorbing boundary, three schemes of ITN coverage are simulated and compared (see Methods for the schemes). No significant difference is found if age-dependent DMRs are used with both boundary types (as mentioned before, using fixed DMRs is not practical for the density-regulated ABM).

While applying LSM and ITNs in combination, some synergistic effects are observed in the results. However, as shown in Figure 11, the combined impact is additive (and not multiplicative), and is more effective with high *density*_*houses*_, confirming similar findings in [2].

With higher *density*_*houses*_, impact of ITN mortality (M) becomes increasingly important. As shown in Figure 11, increasing ITN mortality affects the shape of the low-to-medium range (10-40%) PR isolines. With no insecticidal effect of ITNs (i.e., M = 0.0), looking at row 1 of Additional file 13, as *density*_*houses*_ increases, more host-seeking events occur, causing more mosquitoes to seek for aquatic habitats in order to lay eggs. But with increasing LSM coverage, they are denied more opportunities to lay eggs (as more aquatic habitats are eliminated), causing the lower range (10-40%) PR isolines to reduce vertically (down the y-axis). However, as both *density*_*houses*_ and ITN coverage increase (but mortality still remains 0), more host-seeking events actually encounter ITNs, but with no mortality in effect, ITNs cannot have significant impact, thus extending the lower range (10-40%) PR isolines horizontally (across the x-axis). As ITN mortality increases (in Figure 11 and rows 2-4 of Additional file 13), this extension effect is gradually reduced, and more impact is seen with higher *density*_*houses*_.

Replication of earlier ABMs (that examined the impact of LSM and ITNs in isolation) poses some unique challenges. The unavailability of source codes of the original models inhibits from performing direct model-to-model comparison (docking). The structural characteristics of ABMs, which are fundamentally different from, for example, equation-based mathematical models, also rule out the possibility of systematic verification of model features, and draw some important V&V issues. The following major sources are identified from which model differences may arise, and/or the process of replication may become more time-consuming and challenging:

•Conceptual image of the model: the intended logical view of the ABM may be perceived differently by different modellers, thus creating different conceptual, *mental* images of the logical view.

•Choice of tools: selection of programming languages and tools (e.g., C++ vs. Java) from the numerous options offered these days may be another potential source. The availability and limitations of a particular programming language, use of specific data structures and other language constructs, and even the coding style of individual modellers, can compound the differences.

•Availability of additional resources: in some cases, additional resources used by the model (in the forms of artificial maps, object-based landscapes, etc.), if not defined or made explicitly available, pose subtle challenges. Although the importance of these resources may seem somewhat arbitrary in the broader context, goals and output of the original models, for replication, their precise specification still remains important. For example, as shown before, in replicating the landscapes, the absence of a listing of the spatial coordinates of the objects (which may be provided as supplementary materials), not only forces future modellers who try to replicate the landscapes to spend a significant amount of time in reproducing the landscapes (some part of which inevitably rely on best guesses, due to the lack of additional information), it also increases the possibility of judgement errors being introduced in this phase.

Clear, detailed description of the parameter space for all model parameters used by the ABM, including their initial and other time-varying conditions, may substantially help in minimizing the conceptual image gaps. However, as the past experience shows [19,20], merely stating model parameters, logical flowcharts, initial conditions etc. cannot entirely solve the above problems, primarily because: (1) the possibility of different logical workflow paths in the programmed code still remains open and (2) many implementation details still cannot be covered. Based on this modelling exercise, the following guidelines are recommended for future ABM modellers of malaria:

•Code and data sharing: The source code and executable programs of the ABMs should be shared with the research community. The trends of open-access research have become increasingly important and popular in recent years. To ensure a minimum standard of reproducibility in computational sciences, enough information about methods and code should be available for independent researchers to reach consistent conclusions [37]. Many reputed journals across multiple disciplines have also implemented different code-sharing policies. For example, the journal *Biostatistics*[54] has implemented a policy to encourage authors of accepted papers to make their work reproducible by others. In this journal, based on three different criteria termed as “code”, “data” and “reproducible”, the associate editor for reproducibility (AER) classifies the accepted papers as C, D and/or R, respectively, on their title pages [37,55]. As reproducibility is critical to tracking down the bugs of computational science, code-sharing may be specially important for malaria ABMs. Having multiple research groups examining the same model and generating new data or output may lead to robust conclusions [39]. Some recent malaria models have partially followed this path by providing controlled access to their models. For example, the OpenMalaria epidemiology model [56] provides a general open-access platform for comparing, fitting, and evaluating different model structures. The EMOD vector ecology model, from Intellectual Ventures Lab [57], is available within controlled execution environments. However, for certain reasons (e.g., during preliminary design and development phases, exploratory feature testing phases, etc.), it may not always be the ideal case to share the source code. In these cases, it is recommended that for ABM-based studies which are accepted for publication, at least the associated executable programs and/or other tools be made available as supplementary materials (for this study, the ABM, a sample input file and the landscape generator tool are shared as Additional files 1, 2 and 3, respectively, with detailed instructions on how to run).

•Relevant documentation: Modellers who share the source code and/or executable programs of their ABMs should also provide well-written documentation. Documentation is an important part of software engineering. The journal *PLOS Computational Biology*, which publishes articles describing outstanding open source software, emphasizes that the source code must be accompanied with documentation on building and installing the software from the source, including instructions on how to use and test the software on supplied test data [58]. An ABM documentation may include statements describing the attributes, features and characteristics of the agents and environments of the ABM, the overall architecture or design principles of the code, algorithms and application programming interfaces (APIs), manuals for end-users, interpretation of additional materials (e.g., object-based landscapes), etc. Free and commercial software tools are available which can help automating the process of code annotation, code analysis and software documentation [59-62].

•Standardized models: The general workflow of the ABM, including the input/output requirements, program logic, etc. should follow a standardized approach. The need for standardization becomes more important when the broader utility of the model is considered within an integrated modelling platform. For example, both OpenMalaria [56] and EMOD [57] are currently being integrated within the open-access execution environment of the Vector Ecology and Control Network (VECNet) [63]. The proposed VECNet cyberinfrastructure (VECNet CI), within a shared execution environment, establishes three modes of access sharing for model developers: (1) shared data: model developers run their models on their own compute resources and upload the output data to the VECNet CI for public consumption; (2) shared execution: model developers share their software with VECNet CI developers only, allowing the CI and its operators to incorporate their model into the CI execution environment; and (3) shared software: model developers share their software at large with the public. Once integrated, these models can utilize other components of the VECNet CI, including the VECNet Digital Library, web-based user interface (UI), tools for visualization, job management, query and search, etc. in order to, for example, import and use malaria-specific data to run specific scenarios or campaigns of interest, and display their output using the visualization and/or the UI tools of the VECNet CI. It is envisaged that most malaria ABMs, in future, will be accommodated within the integrated modelling frameworks of similar cyberinfrastructure platforms. Hence, to expedite the integration process, future malaria ABMs should plan and follow a well-defined integration path from the early phases of model development.

## Conclusions

In this study, the individual and combined efficacy of applying LSM and ITNs are explored by using a spatial ABM of malaria that precisely defines the movement rules of adult female mosquitoes in their resource-foraging process in grid-based landscapes. Results of two earlier studies that explored similar research questions [10,11] are replicated, and a systematic comparison of the results are presented. By extending some of the original assumptions (e.g., reporting results from single simulation runs, use of an absorbing boundary, etc.), it is shown that the use of these assumptions may lead to less reliable results. With the combined application of LSM and ITNs, the results indicate that varying densities of the human population can affect the degree of synergistic benefits that may be obtained from such efforts, as was previously shown by a mathematical model [2]. To the best of our knowledge, this is the first ABM-based study to explore this particular combination of LSM and ITNs (acknowledging that some other combinations were explored by other ABMs, e.g., [12]). Some challenges faced while replicating earlier models are also discussed, and several guidelines (code and data sharing, relevant documentation, and standardized models) obtained from this exercise are recommended for future ABM modellers of malaria.

As the results indicate, replicability of the experiments and simulations performed by malaria models published earlier bear special importance. Due to several factors (including new tools and technologies, massive amounts of data, interdisciplinary research, etc.), the task of replication may become complicated. By sharing the ABM and the landscape generator tool, the importance of open source software for reproducibility and replicability is emphasized.

In the future, seasonality and other weather parameters (e.g., humidity), alternative hosts for blood-feeding (e.g., cattle), aquatic habitats with varying carrying capacities to reflect the variability of habitat attractiveness and productivity, and temporal variability for certain intervention parameters (e.g., repellence and insecticidal effect of ITNs) are planned to be included in the model. Calibrating the assumptions and parameters of the model against data from field-based studies, and exploring the impact of other existing interventions (e.g., IRS, space spraying, etc.), or new interventions (e.g., spatial repellents and/or insecticides, oviposition traps, etc.), both in isolation and in combination, are also planned. Lastly, *VectorLand* is planned to be improved to aid in generating operational guidelines for targeting of aquatic habitats and houses, and thus to perform a systematic study of the effect of spatial distribution of habitats.

## Abbreviations

ABM: Agent-based model; ABMS: Agent-based modelling and simulation; ACT: Artemisinin-combination therapy; CC: Carrying capacity; DMR: Daily mortality rate; GN: Gu and Novak; IRS: Indoor residual spraying; ITN: Insecticide-treated net; IVM: Integrated vector management; LLIN: Long-lasting insecticidal net; LSM: Larval source management; MSAT: Mass screening and treatment; PR: Percent reduction; V&V: Verification and validation; VECNet: Vector Ecology and Control Network.

## Competing interests

The authors declare that they have no competing interests.

## Authors’ contributions

SMNA developed the spatial extension of the ABM, performed the literature review for model comparisons, designed the experiments, conducted the simulations, data analysis, replication tasks, and drafted the manuscript. SMNA and GRM interpreted the results. GRM and FHC supervised the study. All authors read and approved the final manuscript.

## Supplementary Material

Additional file 1 **The spatial agent-based model (in JAR format).** The ABM is developed in Java, using the Eclipse SDK (Version: 3.5.2), which is freely available from [64]. To run the JAR file, the computer must have the Java Runtime Environment (which can be downloaded from [65]). Instructions: (1) save the JAR file and the sample input file (Additional file 2) in a directory; (2) navigate into the directory: for Windows, use ‘chdir’ or ‘cd’ in a command prompt; for UNIX/Mac, use ‘cd’ in a terminal; (3) issue the command: *java -Xms1g -Xmx8g -d64 -jar ./Additional file**1**.jar ./Additional file**2**.xml optionalId* where *optionalId* refers to an optional integer (e.g., 123) to sequence different simulation runs. To change parameters, modify the sample input file. The runtime usually ranges a few hours. It primarily depends on the length of the simulation (maximum time-step), width and height of the landscape, number of all aquatic habitats and houses, and carrying capacity for each aquatic habitat, all of which may be specified in the input file. The ABM will create two plain-text output files (under two newly-created directories), both named according to the major parameters (as extracted from the input XML file), and time-stamped with the simulation start-time: (1) the FA.txt file, which contains the number of female adult mosquitoes in the system (one entry per day), and (2) the SimPara.txt file, which contains information about the simulation job, including a listing of the major parameters and simulation events (e.g., application of interventions). For the same input XML file, multiple/batch runs may be conducted by using simple scripts (e.g., UNIX shell scripts). Note that the ABM may be run by supplying hand-generated XML input files (e.g., Additional file 2), or by generating new XML files using *VectorLand* (see Additional file 3). Click here for file

Additional file 2 **Sample input file (in XML format) for the ABM.** The ABM (Additional file 1) requires an XML file as its only input. The XML file can be viewed and edited using any standard text editor (e.g., Notepad for Windows, TextEdit for Mac, etc.). It specifies various parameters of the model, the simulation to be run, the landscape to be used, and the interventions to be applied. Model parameters include length of the simulation (maximum time-step), initial number of female adult mosquitoes, fecundity (mean and standard deviation), initial temperature, and daily mortality rates for the immature (eggs, larvae, pupae) and adult stages of the mosquito life cycle. Landscape parameters include an optional landscape name, width and height of the landscape, the boundary type to be used (Absorbing or Nonabsorbing ), and maximum number of moves allowed for a mosquito agent per day. Simulation parameters specify all aquatic habitats and blood meal locations (houses) as individual sub-environments. Description of each sub-environment includes an optional identifier, its spatial location (x- and y-coordinates), carrying capacity (for an aquatic habitat), number of persons (for a blood meal location), and whether the sub-environment is covered by LSM or ITNs. Intervention parameters include the beginning time-step to apply the intervention, coverage, and repellence, mortality/insecticidal effect (for ITNs). The current model supports only LSM and ITNs as interventions. This sample file specifies 200 aquatic habitats, 20 houses, 30% LSM coverage, 50% ITN coverage, no repellence, and 70% ITN mortality (insecticidal effect). Click here for file

Additional file 3 **The landscape generator tool *VectorLand* (in ZIP format)*.*** To run *VectorLand*, first unzip the file (Additional file 3.zip). To run the JAR file (the computer must have the Java Runtime Environment, which can be downloaded from [65]), double-click the file (VectorLand.jar). To run from the command line: (1) navigate into the unzipped directory: for Windows, use ‘chdir’ or ‘cd’ in a command prompt; for UNIX/Mac, use ‘cd’ in a terminal; (2) issue the command: java -jar ./VectorLand.jar. *VectorLand* is developed in Java, using the NetBeans IDE (Version: 7.1.1), which is freely available from [66]. Once the VectorLand screen appears, to create a new landscape, modify the desired parameters, and then click the *Update* button (or hit the *Enter* key on keyboard). The spatial distribution of the aquatic habitats and blood meal locations, along both axes, can be controlled using the “Clustering” sliders. A scale of 1 to 10 is used, where 1 means the most clustered, and 10 means the least clustered. To save the landscape, click the *Save* button. All landscapes will be saved in a directory named as *Date-Landscapes* (if the directory does not exist, it would be automatically created; *Date* would be auto-generated as well). The current landscape will be saved as *Version-LandscapeName.xml* (where *Version* refers to an auto-generated version number, and *LandscapeName* refers to the landscape name as displayed at the lower-left of *VectorLand*). A JPEG image of the landscape will also be saved as *Version-LandscapeName.jpg*. Clicking the *Help* button will display a short tutorial. Currently, it supports only 40 × 40 landscapes. Click here for file

Additional file 4 ***VectorLand* screenshot.***VectorLand* can generate landscapes with varying spatial heterogeneity of both types of resources: aquatic habitats and houses (blood meal locations). Locations of resources can be controlled using the *Clustering* sliders across both axes (see earlier version in [19] for details). Intervention parameters can be controlled using separate panels (currently for LSM and ITNs). This screenshot depicts selecting *Medium density*_*houses*_, with 30% LSM coverage and 50% ITN coverage. Additional statistics about the generated landscape and legends are also shown in separate panels. Click here for file

Additional file 5 **The landscapes digitized from the GN-LSM study**[10]. The 40 × 40 grid-based landscapes, digitized and reproduced from the GN-LSM study [10], by using the landscape generator tool, *VectorLand*. Each landscape contains 70 aquatic habitats (blue circles) and 20 houses (black house icons). Within each landscape, the houses are arranged diagonally, horizontally, or vertically. For each arrangement, seven scenarios of LSM are shown; from left to right: NOCTRL (no LSM), T1, T2, T3, C1, C2, C3. T1, T2 and T3 refer to targeted removal of aquatic habitats within 100, 200 and 300 m of surrounding houses, accounting for 4, 17 and 28 of 70 habitats, respectively. C1, C2 and C3 refer to non-targeted, random removal of the same numbers of aquatic habitats as the corresponding targeted interventions. Click here for file

Additional file 6 The landscape digitized from the GN-ITN study [11]. The 40 × 40 grid-based landscape, digitized and reproduced from the GN-ITN study [11], by using the landscape generator tool, *VectorLand*. It contains 90 aquatic habitats (blue circles) that are randomly distributed, and 50 houses (black squares) that are arranged diagonally. (PDF 98 kb) Click here for file

Additional file 9 **Full one-year results showing the impact of ITNs (applied in isolation) on mosquito abundance, using the household-level *partial* coverage scheme with *single *chance for host-seeking.** Each subfigure represents a specific combination of coverage (C) and repellence (R) for ITNs: **(1)** C = 0.4, R = 0.2, **(2)** C = 0.4, R = 0.5, **(3)** C = 0.4, R = 0.9, **(4)** C = 0.6, R = 0.2, **(5)** C = 0.6, R = 0.5, **(6)** C = 0.6, R = 0.9, **(7)** C = 0.8, R = 0.2, **(8)** C = 0.8, R = 0.5, **(9)** C = 0.8, R = 0.9, **(10)** C = 1.0, R = 0.2, **(11)** C = 1.0, R = 0.5 and **(12)** C = 1.0, R = 0.9. Within each subfigure, each colour-coded plot represents a specific mortality (M) value for ITNs (e.g., M = 0.25), with mortality (M) colour keys at the bottom of the figure. The figure represents averages of a total of 3,000 (4 × 3 × 5 × 50) simulations. For other details, see Figure 3(a) and Figure 8. Click here for file

Additional file 10 **Full one-year results showing the impact of ITNs (applied in isolation) on mosquito abundance, using the household-level *partial *coverage scheme with *multiple *chances for host-seeking.** Each subfigure represents a specific combination of coverage (C) and repellence (R) for ITNs: **(1)** C = 0.4, R = 0.2, **(2)** C = 0.4, R = 0.5, **(3)** C = 0.4, R = 0.9, **(4)** C = 0.6, R = 0.2, **(5)** C = 0.6, R = 0.5, **(6)** C = 0.6, R = 0.9, **(7)** C = 0.8, R = 0.2, **(8)** C = 0.8, R = 0.5, **(9)** C = 0.8, R = 0.9, **(10)** C = 1.0, R = 0.2, **(11)** C = 1.0, R = 0.5 and **(12)** C = 1.0, R = 0.9. Within each subfigure, each colour-coded plot represents a specific mortality (M) value for ITNs (e.g., M = 0.25), with mortality (M) colour keys at the bottom of the figure. The figure represents averages of a total of 3,000 (4 × 3 × 5 × 50) simulations. For other details, see Figure 3(b) and Figure 8. Click here for file

Additional file 11 **Full one-year results showing the impact of ITNs (applied in isolation) on mosquito abundance, using the household-level *complete* coverage scheme.** Each subfigure represents a specific combination of coverage (C) and repellence (R) for ITNs: **(1)** C = 0.4, R = 0.2, **(2)** C = 0.4, R = 0.5, **(3)** C = 0.4, R = 0.9, **(4)** C = 0.6, R = 0.2, **(5)** C = 0.6, R = 0.5, **(6)** C = 0.6, R = 0.9, **(7)** C = 0.8, R = 0.2, **(8)** C = 0.8, R = 0.5, **(9)** C = 0.8, R = 0.9, **(10)** C = 1.0, R = 0.2, **(11)** C = 1.0, R = 0.5 and **(12)** C = 1.0, R = 0.9. Within each subfigure, each colour-coded plot represents a specific mortality (M) value for ITNs (e.g., M = 0.25), with mortality (M) colour keys at the bottom of the figure. The figure represents averages of a total of 3,000 (4 × 3 × 5 × 50) simulations. For other details, see Figure 3(c) and Figure 9. Click here for file

Additional file 12 **Percent reductions in mosquito abundance by ITNs, applied in isolation, comparing household-level partial coverage (with multiple chances for host-seeking) and complete coverage.** The x-axis denotes ITN coverage, and the y-axis denotes ITN mortality. Each subfigure represents a specific combination of coverage scheme (partial or complete) and repellence (R) for ITNs. Subfigures **(1)**-**(4)** represent the *partial* coverage scheme with: **(1)** R = 0.0, **(2)** R = 0.2, **(3)** R = 0.5 and **(4)** R = 0.9. Subfigures **(5)**-**(8)** represent the *complete* coverage scheme with: **(5)** R = 0.0, **(6)** R = 0.2, **(7)** R = 0.5 and **(8)** R = 0.9. ITN is applied at day 100 in the 40 × 40 grid-based landscape (see Additional file 6) with 50 houses having a total human population of 185. The percent reduction (PR) values, represented as filled contour plots in each subfigure, are calculated from data used in Additional files 10 and 11. The colourbar on the right quantifies the PR isolines. (PDF 28 kb) Click here for file

Additional file 13 **Percent reductions in mosquito abundance as a function of LSM coverage and ITN coverage when LSM and ITNs are applied in combination.** The x-axis denotes ITN coverage, and the y-axis denotes LSM coverage. Each subfigure represents a specific combination of density of houses (*density*_*houses*_ with values *Low*, *Medium* and *High*, see Table 4) with mortality (M) for ITNs: subfigures **(1)**-**(3)** represent M = 0.0, subfigures **(4)**-**(6)** represent M = 0.2, subfigures **(7)**-**(9)** represent M = 0.5 and subfigures **(10)**-**(12)** represent M = 0.8. ITN repellence (R) is fixed at 0.5. Each simulation is run for one year; both LSM and ITNs are applied at day 100, and continued up to the end of the simulation. Each subfigure represents filled contour plots where the isolines are labelled with specific percent reduction (PR) values. The colourbar on the right quantifies the PR isolines. The figure represents average percent reduction values of a total of 12,000 (3 × 5 × 4 × 4 × 50) simulations. For ITNs, household-level *complete* coverage scheme is used (see Figure 3c). A non-absorbing boundary is used. Sample landscapes with the three *density*_*houses*_ levels are shown in Figure 4. Click here for file

Additional file 7 **Full one-year results showing the impact of LSM (applied in isolation) on mosquito abundance: a comparison with the GN-LSM study**[10]**using an *absorbing *boundary.** Each subfigure represents a specific LSM scenario. Subfigures **(1)**-**(3)**, denoted as C1, C2 and C3, refer to the non-targeted, random removal of the aquatic habitats. Subfigures **(4)**-**(6)**, denoted as T1, T2 and T3, refer to the targeted removal of aquatic habitats within 100, 200 and 300 m of surrounding houses. The non-targeted scenarios remove the same numbers of aquatic habitats as in the corresponding targeted scenarios (for example, both C1 and T1 remove 4 habitats). For details about the LSM scenarios used in the subfigures, see legend of Figure 5. Click here for file

Additional file 8 **Full one-year results showing the impact of LSM (applied in isolation) on mosquito abundance: a comparison with the GN-LSM study**[10]** using a *non-absorbing *boundary.** Each subfigure represents a specific LSM scenario. Subfigures **(1)**-**(3)**, denoted as C1, C2 and C3, refer to the non-targeted, random removal of aquatic habitats. Subfigures **(4)**-**(6)**, denoted as T1, T2 and T3, refer to the targeted removal of aquatic habitats within 100, 200 and 300 m of surrounding houses. For details about the LSM scenarios used in the subfigures, see legend of Figure 6. Click here for file
