# Supplementary figures and images for: Examining the impact of larval source management and insecticide-treated nets using a spatial agent-based model of Anopheles gambiae and a landscape generator tool
Source: Malar J. 2013 Aug 21;12:290. doi: 10.1186/1475-2875-12-290 (PMC3765353; doi:10.1186/1475-2875-12-290)

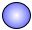

Supplement: Additional file 3 — The landscape generator tool VectorLand (in ZIP format). To run VectorLand, first unzip the file (Additional file 3.zip). To run the JAR file (the computer must have the Java Runtime Environment, which can be downloaded from [65]), double-click the file (VectorLand.jar). To run from the command line: (1) navigate into the unzipped directory: for Windows, use ‘chdir’ or ‘cd’ in a command prompt; for UNIX/Mac, use ‘cd’ in a terminal; (2) issue the command: java -jar ./VectorLand.jar. VectorLand is developed in Java, using the NetBeans IDE (Version: 7.1.1), which is freely available from [66]. Once the VectorLand screen appears, to create a new landscape, modify the desired parameters, and then click the Update button (or hit the Enter key on keyboard). The spatial distribution of the aquatic habitats and blood meal locations, along both axes, can be controlled using the “Clustering” sliders. A scale of 1 to 10 is used, where 1 means the most clustered, and 10 means the least clustered. To save the landscape, click the Save button. All landscapes will be saved in a directory named as Date-Landscapes (if the directory does not exist, it would be automatically created; Date would be auto-generated as well). The current landscape will be saved as Version-LandscapeName.xml (where Version refers to an auto-generated version number, and LandscapeName refers to the landscape name as displayed at the lower-left of VectorLand). A JPEG image of the landscape will also be saved as Version-LandscapeName.jpg. Clicking the Help button will display a short tutorial. Currently, it supports only 40 × 40 landscapes. [file 1475-2875-12-290-S3.zip › VectorLand/images/aH.png]

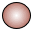

Supplement: Additional file 3 — The landscape generator tool VectorLand (in ZIP format). To run VectorLand, first unzip the file (Additional file 3.zip). To run the JAR file (the computer must have the Java Runtime Environment, which can be downloaded from [65]), double-click the file (VectorLand.jar). To run from the command line: (1) navigate into the unzipped directory: for Windows, use ‘chdir’ or ‘cd’ in a command prompt; for UNIX/Mac, use ‘cd’ in a terminal; (2) issue the command: java -jar ./VectorLand.jar. VectorLand is developed in Java, using the NetBeans IDE (Version: 7.1.1), which is freely available from [66]. Once the VectorLand screen appears, to create a new landscape, modify the desired parameters, and then click the Update button (or hit the Enter key on keyboard). The spatial distribution of the aquatic habitats and blood meal locations, along both axes, can be controlled using the “Clustering” sliders. A scale of 1 to 10 is used, where 1 means the most clustered, and 10 means the least clustered. To save the landscape, click the Save button. All landscapes will be saved in a directory named as Date-Landscapes (if the directory does not exist, it would be automatically created; Date would be auto-generated as well). The current landscape will be saved as Version-LandscapeName.xml (where Version refers to an auto-generated version number, and LandscapeName refers to the landscape name as displayed at the lower-left of VectorLand). A JPEG image of the landscape will also be saved as Version-LandscapeName.jpg. Clicking the Help button will display a short tutorial. Currently, it supports only 40 × 40 landscapes. [file 1475-2875-12-290-S3.zip › VectorLand/images/aHToBeRemoved.png]

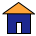

Supplement: Additional file 3 — The landscape generator tool VectorLand (in ZIP format). To run VectorLand, first unzip the file (Additional file 3.zip). To run the JAR file (the computer must have the Java Runtime Environment, which can be downloaded from [65]), double-click the file (VectorLand.jar). To run from the command line: (1) navigate into the unzipped directory: for Windows, use ‘chdir’ or ‘cd’ in a command prompt; for UNIX/Mac, use ‘cd’ in a terminal; (2) issue the command: java -jar ./VectorLand.jar. VectorLand is developed in Java, using the NetBeans IDE (Version: 7.1.1), which is freely available from [66]. Once the VectorLand screen appears, to create a new landscape, modify the desired parameters, and then click the Update button (or hit the Enter key on keyboard). The spatial distribution of the aquatic habitats and blood meal locations, along both axes, can be controlled using the “Clustering” sliders. A scale of 1 to 10 is used, where 1 means the most clustered, and 10 means the least clustered. To save the landscape, click the Save button. All landscapes will be saved in a directory named as Date-Landscapes (if the directory does not exist, it would be automatically created; Date would be auto-generated as well). The current landscape will be saved as Version-LandscapeName.xml (where Version refers to an auto-generated version number, and LandscapeName refers to the landscape name as displayed at the lower-left of VectorLand). A JPEG image of the landscape will also be saved as Version-LandscapeName.jpg. Clicking the Help button will display a short tutorial. Currently, it supports only 40 × 40 landscapes. [file 1475-2875-12-290-S3.zip › VectorLand/images/bML.png]

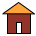

Supplement: Additional file 3 — The landscape generator tool VectorLand (in ZIP format). To run VectorLand, first unzip the file (Additional file 3.zip). To run the JAR file (the computer must have the Java Runtime Environment, which can be downloaded from [65]), double-click the file (VectorLand.jar). To run from the command line: (1) navigate into the unzipped directory: for Windows, use ‘chdir’ or ‘cd’ in a command prompt; for UNIX/Mac, use ‘cd’ in a terminal; (2) issue the command: java -jar ./VectorLand.jar. VectorLand is developed in Java, using the NetBeans IDE (Version: 7.1.1), which is freely available from [66]. Once the VectorLand screen appears, to create a new landscape, modify the desired parameters, and then click the Update button (or hit the Enter key on keyboard). The spatial distribution of the aquatic habitats and blood meal locations, along both axes, can be controlled using the “Clustering” sliders. A scale of 1 to 10 is used, where 1 means the most clustered, and 10 means the least clustered. To save the landscape, click the Save button. All landscapes will be saved in a directory named as Date-Landscapes (if the directory does not exist, it would be automatically created; Date would be auto-generated as well). The current landscape will be saved as Version-LandscapeName.xml (where Version refers to an auto-generated version number, and LandscapeName refers to the landscape name as displayed at the lower-left of VectorLand). A JPEG image of the landscape will also be saved as Version-LandscapeName.jpg. Clicking the Help button will display a short tutorial. Currently, it supports only 40 × 40 landscapes. [file 1475-2875-12-290-S3.zip › VectorLand/images/bMLWithITN.png]

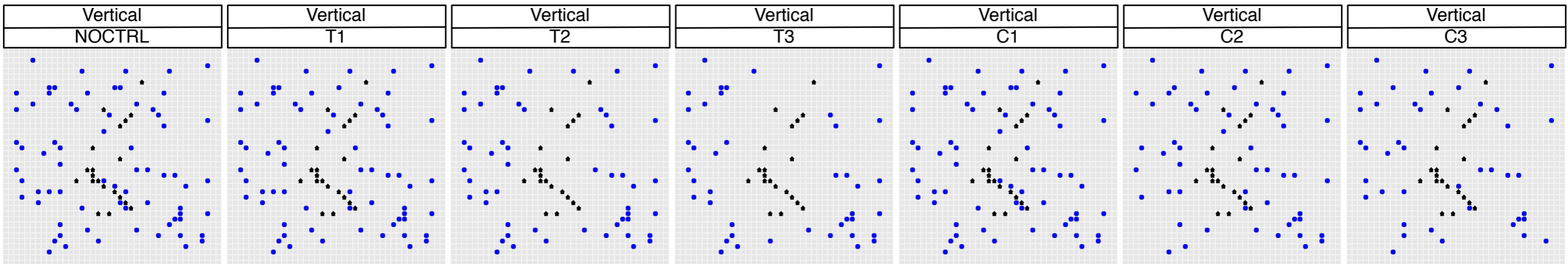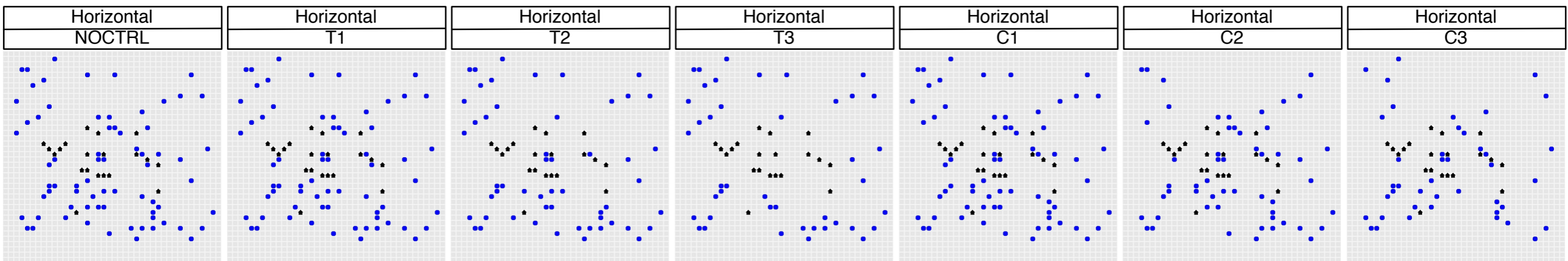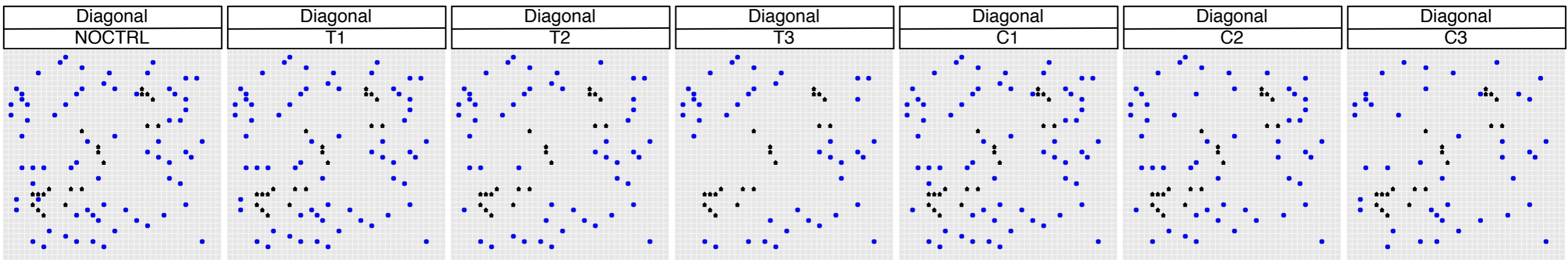

Supplement: Additional file 5 — The landscapes digitized from the GN-LSM study[10]. The 40 × 40 grid-based landscapes, digitized and reproduced from the GN-LSM study [10], by using the landscape generator tool, VectorLand. Each landscape contains 70 aquatic habitats (blue circles) and 20 houses (black house icons). Within each landscape, the houses are arranged diagonally, horizontally, or vertically. For each arrangement, seven scenarios of LSM are shown; from left to right: NOCTRL (no LSM), T1, T2, T3, C1, C2, C3. T1, T2 and T3 refer to targeted removal of aquatic habitats within 100, 200 and 300 m of surrounding houses, accounting for 4, 17 and 28 of 70 habitats, respectively. C1, C2 and C3 refer to non-targeted, random removal of the same numbers of aquatic habitats as the corresponding targeted interventions. [file 1475-2875-12-290-S5.pdf]

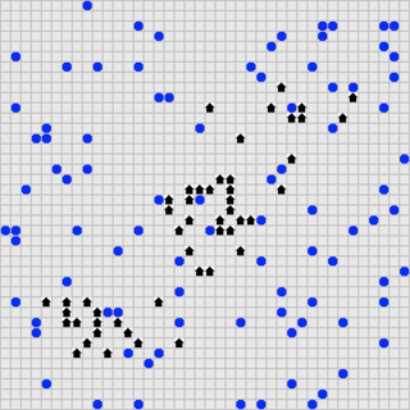

Supplement: Additional file 6 — The landscape digitized from the GN-ITN study [11]. The 40 × 40 grid-based landscape, digitized and reproduced from the GN-ITN study [11], by using the landscape generator tool, VectorLand. It contains 90 aquatic habitats (blue circles) that are randomly distributed, and 50 houses (black squares) that are arranged diagonally. (PDF 98 kb) [file 1475-2875-12-290-S6.pdf]

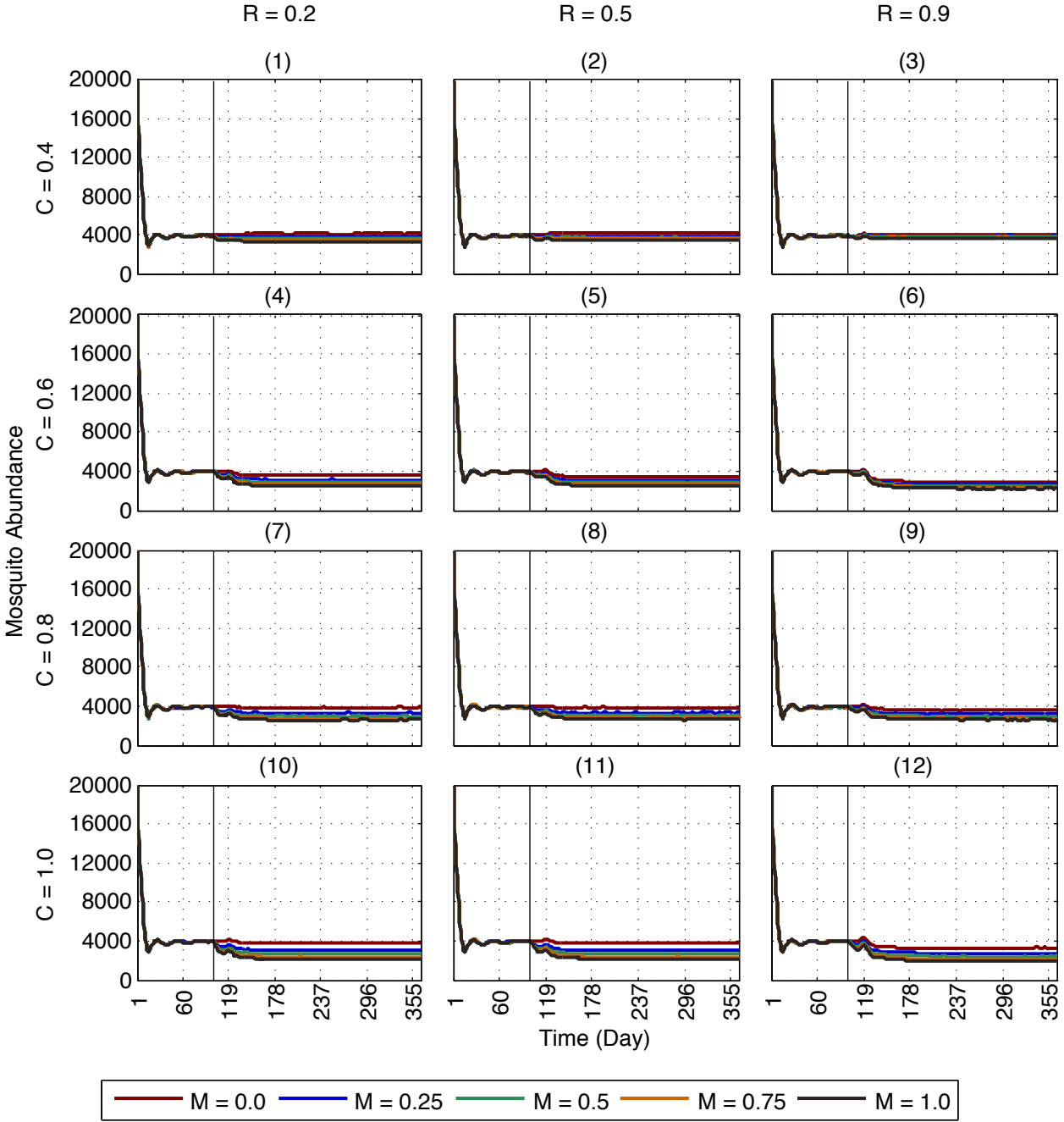

Supplement: Additional file 9 — Full one-year results showing the impact of ITNs (applied in isolation) on mosquito abundance, using the household-level partial coverage scheme with single chance for host-seeking. Each subfigure represents a specific combination of coverage (C) and repellence (R) for ITNs: (1) C = 0.4, R = 0.2, (2) C = 0.4, R = 0.5, (3) C = 0.4, R = 0.9, (4) C = 0.6, R = 0.2, (5) C = 0.6, R = 0.5, (6) C = 0.6, R = 0.9, (7) C = 0.8, R = 0.2, (8) C = 0.8, R = 0.5, (9) C = 0.8, R = 0.9, (10) C = 1.0, R = 0.2, (11) C = 1.0, R = 0.5 and (12) C = 1.0, R = 0.9. Within each subfigure, each colour-coded plot represents a specific mortality (M) value for ITNs (e.g., M = 0.25), with mortality (M) colour keys at the bottom of the figure. The figure represents averages of a total of 3,000 (4 × 3 × 5 × 50) simulations. For other details, see Figure 3(a) and Figure 8. [file 1475-2875-12-290-S9.pdf]

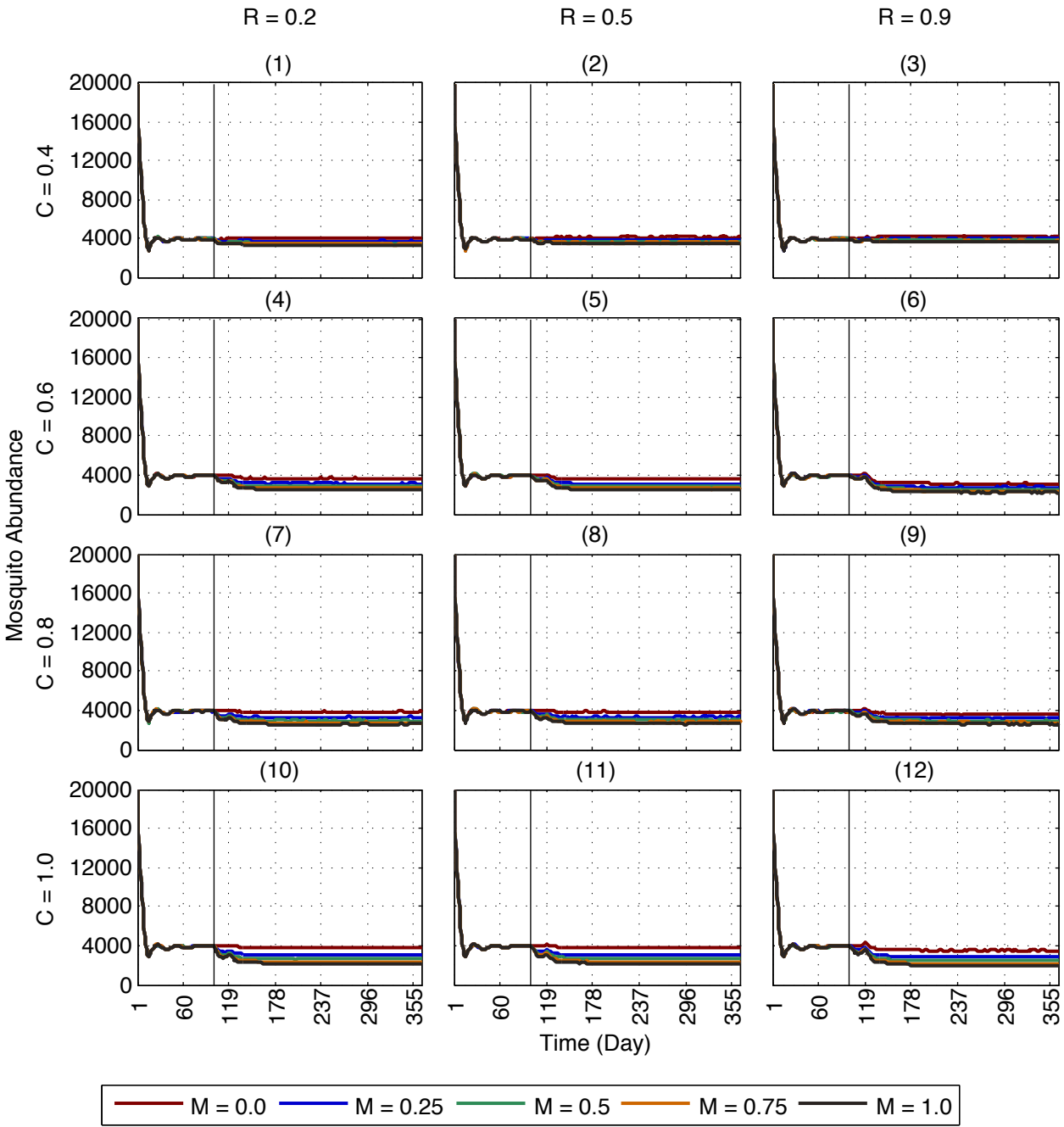

Supplement: Additional file 10 — Full one-year results showing the impact of ITNs (applied in isolation) on mosquito abundance, using the household-level partial coverage scheme with multiple chances for host-seeking. Each subfigure represents a specific combination of coverage (C) and repellence (R) for ITNs: (1) C = 0.4, R = 0.2, (2) C = 0.4, R = 0.5, (3) C = 0.4, R = 0.9, (4) C = 0.6, R = 0.2, (5) C = 0.6, R = 0.5, (6) C = 0.6, R = 0.9, (7) C = 0.8, R = 0.2, (8) C = 0.8, R = 0.5, (9) C = 0.8, R = 0.9, (10) C = 1.0, R = 0.2, (11) C = 1.0, R = 0.5 and (12) C = 1.0, R = 0.9. Within each subfigure, each colour-coded plot represents a specific mortality (M) value for ITNs (e.g., M = 0.25), with mortality (M) colour keys at the bottom of the figure. The figure represents averages of a total of 3,000 (4 × 3 × 5 × 50) simulations. For other details, see Figure 3(b) and Figure 8. [file 1475-2875-12-290-S10.pdf]

$R = 0.2$  $R = 0.5$  $R = 0.9$ 

Mosquito Abundance

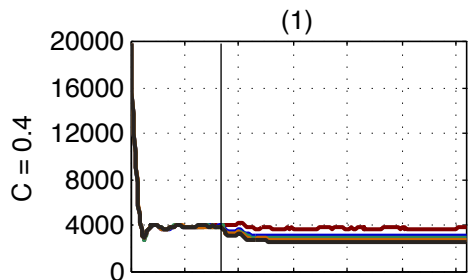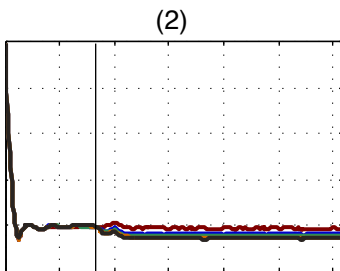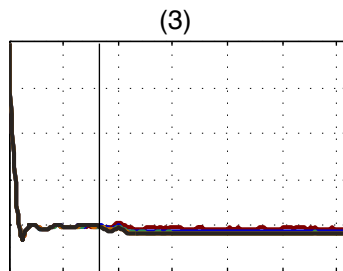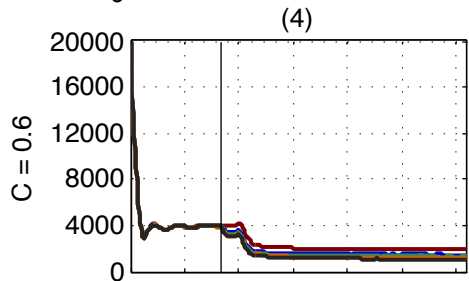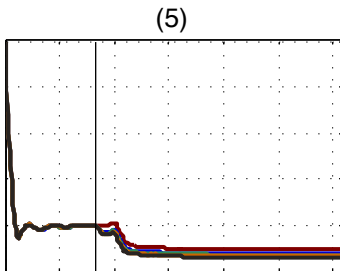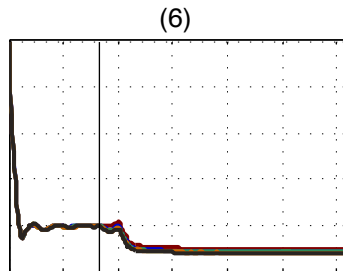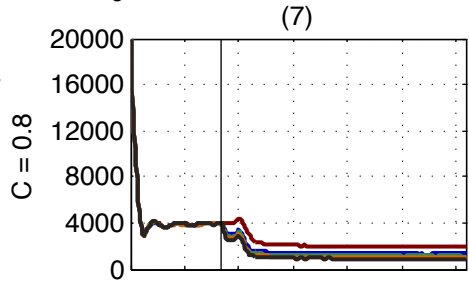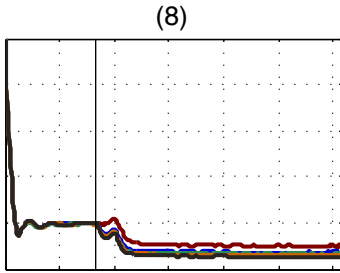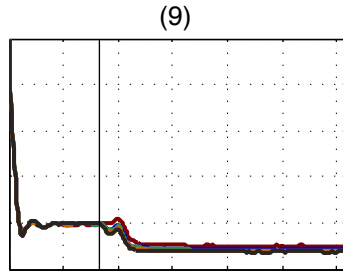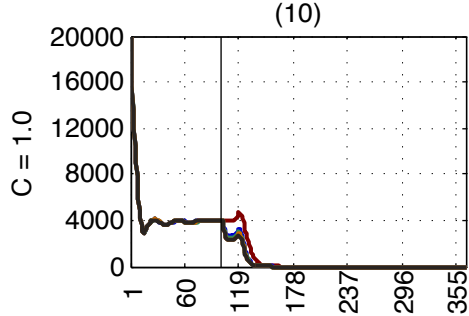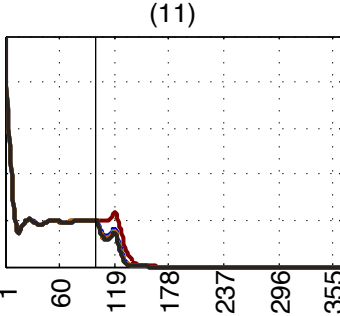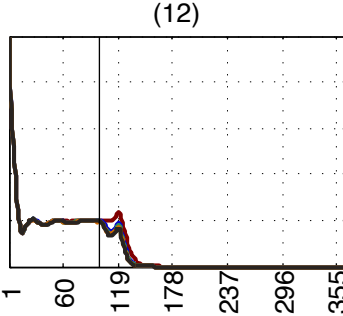

Time (Day)

—  $M = 0.0$  —  $M = 0.25$  —  $M = 0.5$  —  $M = 0.75$  —  $M = 1.0$

Supplement: Additional file 11 — Full one-year results showing the impact of ITNs (applied in isolation) on mosquito abundance, using the household-level complete coverage scheme. Each subfigure represents a specific combination of coverage (C) and repellence (R) for ITNs: (1) C = 0.4, R = 0.2, (2) C = 0.4, R = 0.5, (3) C = 0.4, R = 0.9, (4) C = 0.6, R = 0.2, (5) C = 0.6, R = 0.5, (6) C = 0.6, R = 0.9, (7) C = 0.8, R = 0.2, (8) C = 0.8, R = 0.5, (9) C = 0.8, R = 0.9, (10) C = 1.0, R = 0.2, (11) C = 1.0, R = 0.5 and (12) C = 1.0, R = 0.9. Within each subfigure, each colour-coded plot represents a specific mortality (M) value for ITNs (e.g., M = 0.25), with mortality (M) colour keys at the bottom of the figure. The figure represents averages of a total of 3,000 (4 × 3 × 5 × 50) simulations. For other details, see Figure 3(c) and Figure 9. [file 1475-2875-12-290-S11.pdf]

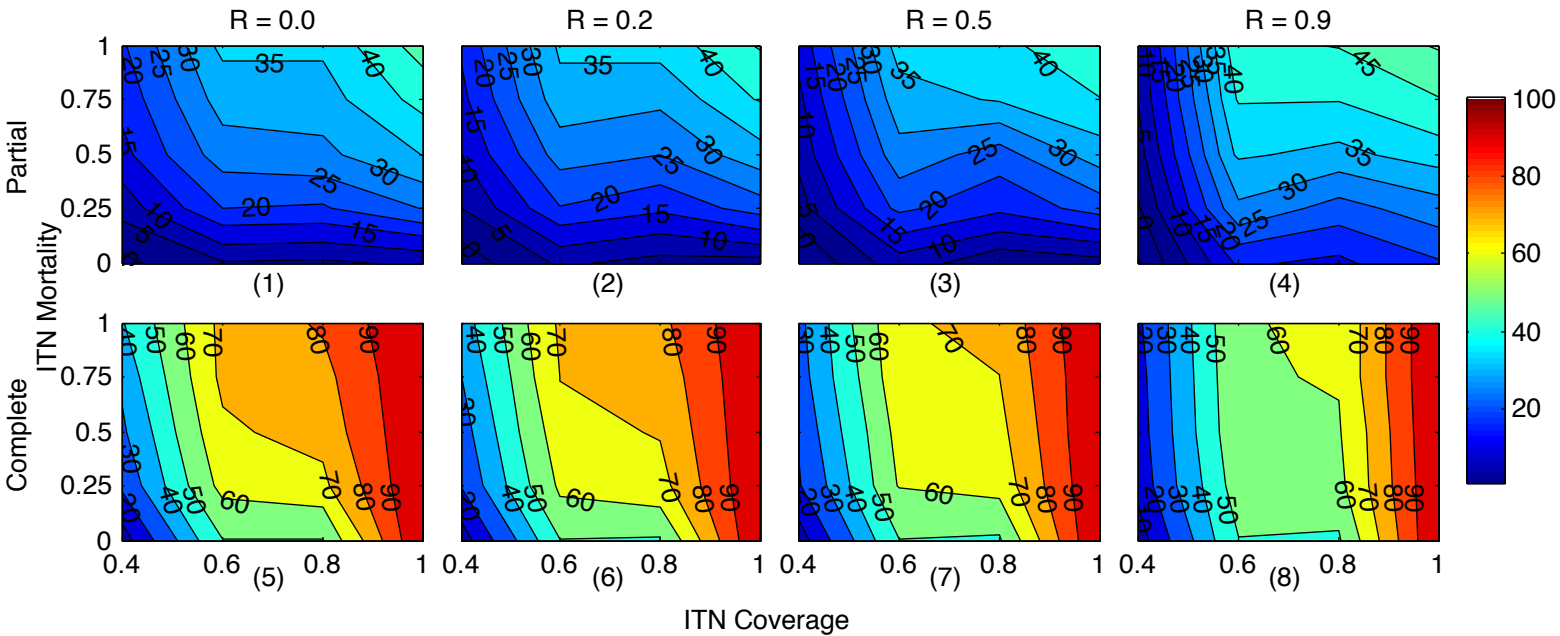

Supplement: Additional file 12 — Percent reductions in mosquito abundance by ITNs, applied in isolation, comparing household-level partial coverage (with multiple chances for host-seeking) and complete coverage. The x-axis denotes ITN coverage, and the y-axis denotes ITN mortality. Each subfigure represents a specific combination of coverage scheme (partial or complete) and repellence (R) for ITNs. Subfigures (1)-(4) represent the partial coverage scheme with: (1) R = 0.0, (2) R = 0.2, (3) R = 0.5 and (4) R = 0.9. Subfigures (5)-(8) represent the complete coverage scheme with: (5) R = 0.0, (6) R = 0.2, (7) R = 0.5 and (8) R = 0.9. ITN is applied at day 100 in the 40 × 40 grid-based landscape (see Additional file 6) with 50 houses having a total human population of 185. The percent reduction (PR) values, represented as filled contour plots in each subfigure, are calculated from data used in Additional files 10 and 11. The colourbar on the right quantifies the PR isolines. (PDF 28 kb) [file 1475-2875-12-290-S12.pdf]

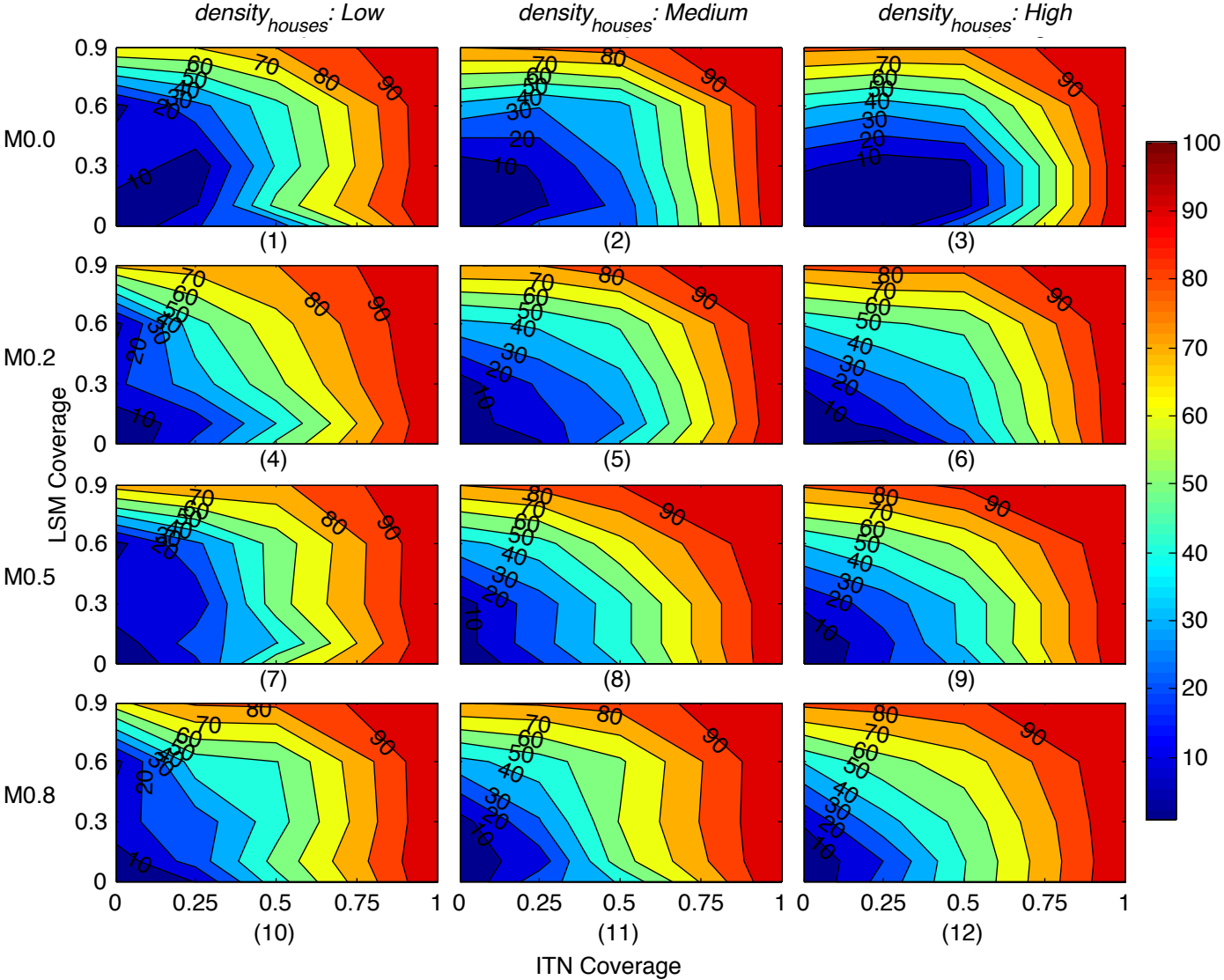

Supplement: Additional file 13 — Percent reductions in mosquito abundance as a function of LSM coverage and ITN coverage when LSM and ITNs are applied in combination. The x-axis denotes ITN coverage, and the y-axis denotes LSM coverage. Each subfigure represents a specific combination of density of houses (densityhouses with values Low, Medium and High, see Table 4) with mortality (M) for ITNs: subfigures (1)-(3) represent M = 0.0, subfigures (4)-(6) represent M = 0.2, subfigures (7)-(9) represent M = 0.5 and subfigures (10)-(12) represent M = 0.8. ITN repellence (R) is fixed at 0.5. Each simulation is run for one year; both LSM and ITNs are applied at day 100, and continued up to the end of the simulation. Each subfigure represents filled contour plots where the isolines are labelled with specific percent reduction (PR) values. The colourbar on the right quantifies the PR isolines. The figure represents average percent reduction values of a total of 12,000 (3 × 5 × 4 × 4 × 50) simulations. For ITNs, household-level complete coverage scheme is used (see Figure 3c). A non-absorbing boundary is used. Sample landscapes with the three densityhouses levels are shown in Figure 4. [file 1475-2875-12-290-S13.pdf]

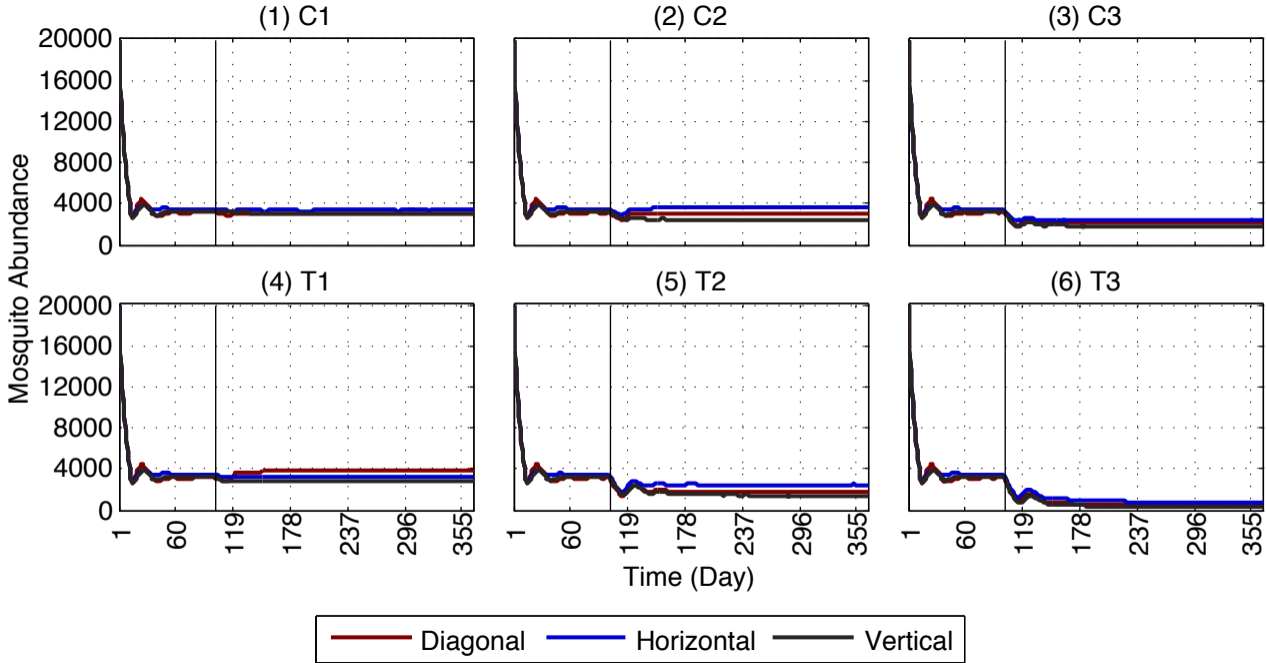

Supplement: Additional file 7 — Full one-year results showing the impact of LSM (applied in isolation) on mosquito abundance: a comparison with the GN-LSM study[10]using an absorbing boundary. Each subfigure represents a specific LSM scenario. Subfigures (1)-(3), denoted as C1, C2 and C3, refer to the non-targeted, random removal of the aquatic habitats. Subfigures (4)-(6), denoted as T1, T2 and T3, refer to the targeted removal of aquatic habitats within 100, 200 and 300 m of surrounding houses. The non-targeted scenarios remove the same numbers of aquatic habitats as in the corresponding targeted scenarios (for example, both C1 and T1 remove 4 habitats). For details about the LSM scenarios used in the subfigures, see legend of Figure 5. [file 1475-2875-12-290-S7.pdf]

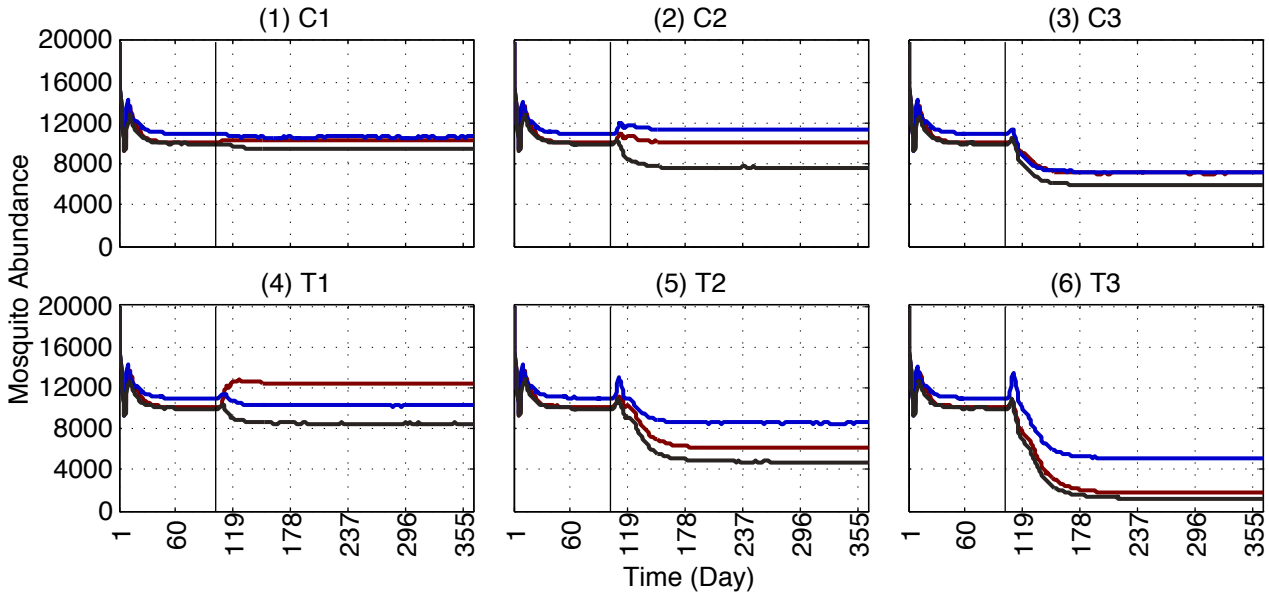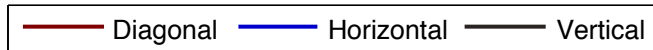

Supplement: Additional file 8 — Full one-year results showing the impact of LSM (applied in isolation) on mosquito abundance: a comparison with the GN-LSM study[10] using a non-absorbing boundary. Each subfigure represents a specific LSM scenario. Subfigures (1)-(3), denoted as C1, C2 and C3, refer to the non-targeted, random removal of aquatic habitats. Subfigures (4)-(6), denoted as T1, T2 and T3, refer to the targeted removal of aquatic habitats within 100, 200 and 300 m of surrounding houses. For details about the LSM scenarios used in the subfigures, see legend of Figure 6. [file 1475-2875-12-290-S8.pdf]
