# Supplementary material for: Examining the impact of larval source management and insecticide-treated nets using a spatial agent-based model of Anopheles gambiae and a landscape generator tool
Source: Malar J. 2013 Aug 21;12:290. doi: 10.1186/1475-2875-12-290 (PMC3765353; doi:10.1186/1475-2875-12-290)
Supplement: Additional file 4 — VectorLand screenshot.VectorLand can generate landscapes with varying spatial heterogeneity of both types of resources: aquatic habitats and houses (blood meal locations). Locations of resources can be controlled using the Clustering sliders across both axes (see earlier version in [19] for details). Intervention parameters can be controlled using separate panels (currently for LSM and ITNs). This screenshot depicts selecting Medium densityhouses, with 30% LSM coverage and 50% ITN coverage. Additional statistics about the generated landscape and legends are also shown in separate panels. [file 1475-2875-12-290-S4.pdf]

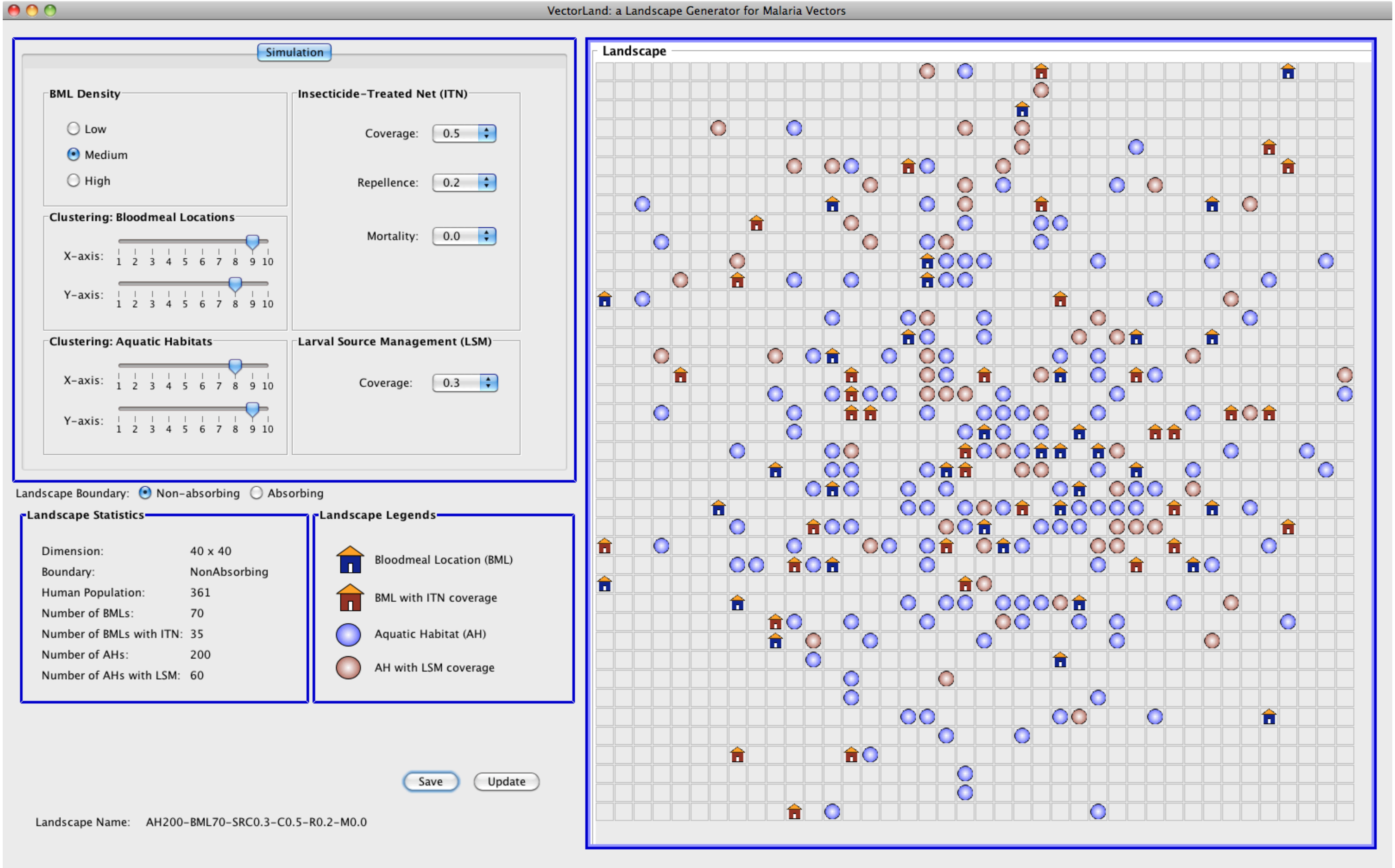
VectorLand: a Landscape Generator for Malaria Vectors
Simulation
BML Density
Low
Medium
High
Insecticide-Treated Net (ITN)
Coverage: 0.5
Repellence: 0.2
Mortality: 0.0
Clustering: Bloodmeal Locations
X-axis: 1 2 3 4 5 6 7 8 9 10
Y-axis: 1 2 3 4 5 6 7 8 9 10
Clustering: Aquatic Habitats
X-axis: 1 2 3 4 5 6 7 8 9 10
Y-axis: 1 2 3 4 5 6 7 8 9 10
Larval Source Management (LSM)
Coverage: 0.3
Landscape Boundary: Non-absorbing Absorbing
Landscape Statistics
Dimension: 40 x 40
Boundary: NonAbsorbing
Human Population: 361
Number of BMLs: 70
Number of BMLs with ITN: 35
Number of AHs: 200
Number of AHs with LSM: 60
Landscape Legends
Bloodmeal Location (BML)
BML with ITN coverage
Aquatic Habitat (AH)
AH with LSM coverage
Save
Update
Landscape Name: AH200-BML70-SRC0.3-C0.5-R0.2-M0.0
Landscape
